# Tedaniophorbasins A and B—Novel Fluorescent Pteridine Alkaloids Incorporating a Thiomorpholine from the Sponge *Tedaniophorbas ceratosis*

**DOI:** 10.3390/md19020095

**Published:** 2021-02-07

**Authors:** Asadhawut Hiranrat, Darren C. Holland, Wilawan Mahabusarakam, John N. A. Hooper, Vicky M. Avery, Anthony R. Carroll

**Affiliations:** 1 School of Environment and Science, Griffith University, Gold Coast, QLD 4222, Australia; hasadhawut@tsu.ac.th (A.H.); darren.holland@griffithuni.edu.au (D.C.H.); 2 Department of Chemistry, Faculty of Science, Prince of Songkla University, Hat Yai, Songkhla 90112, Thailand; wilawan.m@psu.ac.th; 3 Natural Products Research Center, Faculty of Science, Prince of Songkla University, Hat Yai, Songkhla 90112, Thailand; 4 Griffith Institute for Drug Discovery Institute, Griffith University, Brisbane, QLD 4111, Australia; john.hooper@qm.qld.gov.au (J.N.A.H.); v.avery@griffith.edu.au (V.M.A.); 5 Queensland Centre for Biodiversity, Queensland Museum, South Brisbane, QLD 4101, Australia; 6 Discovery Biology, Griffith University, Nathan, QLD 4111, Australia

**Keywords:** sponge, *Tedaniophorbas ceratosis*, pteridine alkaloids, fluorescence, tedaniophorbasin A, malaria

## Abstract

Two new fluorescent pteridine alkaloids, tedaniophorbasins A (**1**) and B (**2**), together with the known alkaloid *N*-methyltryptamine, were isolated, through application of mass directed purification, from the sponge *Tedaniophorbas ceratosis* collected from northern New South Wales, Australia. The structures of tedaniophorbasins A and B were deduced from the analysis of 1D/2D NMR and MS data and through application of ^13^C NMR DFT calculations. Tedaniophorbasin A possesses a novel 2-imino-1,3-dimethyl-2,3,7,8-tetrahydro-1H-[1,4]thiazino[3,2-g]pteridin-4(6*H*)-one skeleton, while tedaniophorbasin B is its 2-oxo derivative. The compounds show significant Stokes shifts (~14,000 cm^−1^) between excitation and emission wavelengths in their fluorescence spectra. The new compounds were tested for bioactivity against chloroquine-sensitive and chloroquine-resistant strains of the malaria parasite *Plasmodium falciparum*, breast and pancreatic cancer cell lines, and the protozoan parasite *Trypanosoma brucei brucei* but were inactive against all targets at 40 µM.

## 1. Introduction

**Figure 1:**
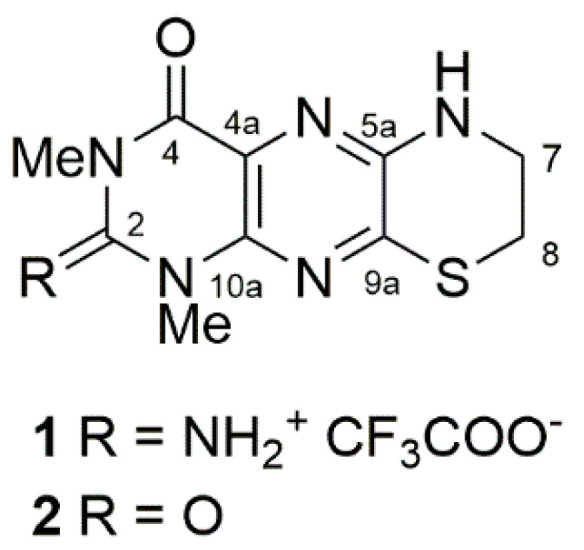
Chemical structures of tedaniophorbasin A (**1**) and B (**2**) isolated from the Australian sponge *Tedaniophorbas ceratosis.*

Chemical diversity has been correlated with biological activity, and the structural diversity of natural products is more like that observed in drugs compared to synthetic libraries [1]. It has also been well documented that the marine environment is a source of unique structural motifs or scaffolds absent in species from the terrestrial environment or in synthetic libraries [2,3]. These novel scaffolds also contain a higher percentage of nitrogen atoms relative to carbon and oxygen compared to scaffolds derived from terrestrial sources [4]. This makes the marine environment a preferred source for biodiscovery. The northern coast of New South Wales in eastern Australia is a biogeographic transition zone in the south-west Pacific that is represented by marine species from both tropical and temperate regions [5]. Little is known about the sessile marine invertebrate (apart from corals) biodiversity of this region, and consequently there has been very little natural product chemistry reported for species collected from this location [6]. Due to the paucity of chemical data reported for species from this region, we have initiated a project to investigate the chemical diversity in relation to the sessile marine invertebrate biodiversity of the region. To date, we have collected over 500 sponges, ascidian, and bryozoan specimens from reefs from this region, and a systematic analysis of the chemistry they contain has been initiated [7,8,9,10,11,12]. Herein, we report on the isolation, structure determination, and biological activity of two new alkaloids, tedaniophorbasins A (**1**) and B (**2**), possessing a novel skeleton (Figure 1) that we have isolated from *Tedaniophorbas ceratosis* collected from oceanic waters adjacent to Cook Island in far northern New South Wales, Australia. This is the first report on the chemistry of sponges from the genus *Tedaniophorbas*.

## 2. Results and Discussion

The frozen sponge was chopped into small pieces and extracted by repeated sonication in MeOH. The MeOH extracts were combined and evaporated, and the yellow brown residue was adsorbed onto C_18_ silica gel. The extract-impregnated gel was separated by preparative HPLC on C_18_-bonded silica gel with a gradient from 1% aqueous TFA to MeOH containing 1% TFA with one min. timed fractions collected. These fractions were further analyzed by (+)−LRESIMS, and only two groups of fractions (fraction 17–20 and fraction 27) showed prominent ions by MS. Fractions 17 and 18 had ion peaks at *m/z* 175 and 265, fractions 19 and 20 had an ion peak at *m/z* 175 and fraction 27 had an ion peak at *m/z* 266. These fractions were evaporated and analyzed by ^1^H NMR spectroscopy. Fractions 17 and 18 contained a mixture of **1** and *N*-methyltryptamine. These compounds were separated from each other by recrystallization from MeOH. The supernatant was pure *N*-methyltryptamine, and **1** was obtained as fine needles (18.5 mg, 0.09%). Fractions 19 and 20 were pure *N*-methyltryptamine (34.7 mg, 0.17%) and fraction 27 was pure **2** (1.3 mg, 0.007%).

**Figure 2:**
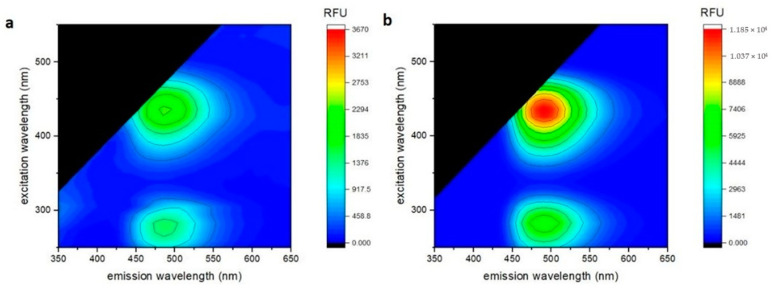
3D fluorescence spectra of tedaniophorbasins A (**a**) and B (**b**).

Tedaniophorbasin A (**1**) was isolated as fluorescent yellow needles as its TFA salt. Positive HRESIMS measurement of the MH^+^ peak (at *m/z* 265.0863 (Δ −1.2 ppm)) established a molecular formula of C_10_H_12_N_6_OS for **1**, inferring that it contained eight degrees of unsaturation. The IR spectrum of **1** had absorption bands at 1712 and 1679 cm^−1^, suggesting that it contained amide and aromatic groups. The UV spectrum had absorption maxima at 223, 280, 296, and 419 nm, indicating that **1** contained an extended aromatic chromophore. Excitation at the absorption maxima at 280 and 419 nm led to intense fluorescence at 490 nm (Figure 2).

The ^1^H NMR spectrum of **1** (Table 1) was very simple, with only six resonances being observed. Edited HSQC correlations (Figure S5) indicated that two of the proton singlets could be assigned to *N*-methyl groups δ_H/C_ 3.66/31.3 and 3.46/30.3 and two were methylene groups δ_H/C_ 3.65/40.6 and 3.34/26.3, while the remaining two signals were due to protons that were not attached to carbons. COSY correlations (Figure S4) between the methylene resonance at δ_H_ 3.65 and both the methylene resonance at δ_H_ 3.34 and an amine signal at δ_H_ 8.15 indicated that a CH_2_CH_2_NH moiety was present in the molecule. The ^1^H and ^13^C chemical shifts for the methylene at 3.34/26.3 were consistent with it being substituted by a sulfur atom [13].

The ^13^C NMR spectrum (Table 1, Figure S3) contained six additional signals not observed in the HSQC spectrum, and these could all be assigned to non-protonated sp^2^ carbons. HMBC correlations (Figures S6–S8) from the *N*-methyl proton resonance at δ_H_ 3.66 to carbons at δ_C_ 151.0 and 137.6 and from the *N*-methyl proton resonance at δ_H_ 3.46 to carbons at δ_C_ 151.0 and 157.4 in combination with the presence of a two-proton broad singlet at δ_H_ 9.05 suggested that a 1,3-di-*N*-methylpyrimidin-2-imino-4-one moiety was present in the molecule. HMBC correlations from the two methylene proton resonances and the amine proton at δ_H_ 8.15 to the carbon resonances at δ_C_ 147.9/148.0 suggested that a disubstituted dehydrothiomorpholine was present in the molecule. The chemical shift of the remaining carbon signal at δ_C_ 120.6 was consistent with it being assigned to C-4a of the pyrimidine [13].

**Figure 3:**
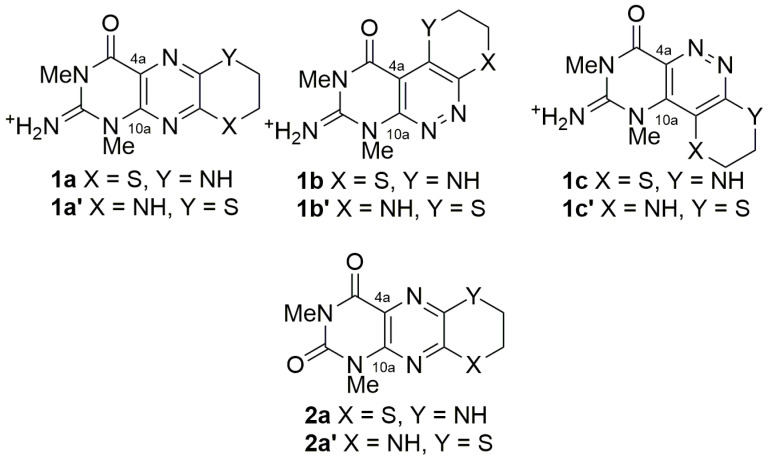
Six alternative structures for tedaniophorbasin A and two alternative structures for tedaniophorbasin B.

The degree of unsaturation derived from the molecular formula required two additional sp^2^ nitrogen atoms to be present in the molecule. These atoms could only logically be placed between the pyrimidine and thiomorpholine partial structures, and six alternative structures (two pyrazine (**1a**/**1a′**) and four pyridazines (**1b**/**1b′** and **1c**/**1c′**) (Figure 3)) could be proposed, with three pairs of regioisomers about the thiomorpholino moiety being possible. Based on the observed chemical shifts, the four pyridazine structures were rejected. In the two pyridazine structures containing a C-C bond between C-4a and the thiomorpholine (structures **1b**/**1b′**), C-4a is predicted to be shielded and resonate at ~ δ_C_ 110 and C-10a deshielded, resonating at ~δ_C_ 160, while in the two pyridazine structures with a C-C bond between C-10a and the thiomorpholino moiety (structures **1c**/**1c′**), C-4a and C-10a would both resonate at ~δ_C_ 135 [13].

**Figure 4:**
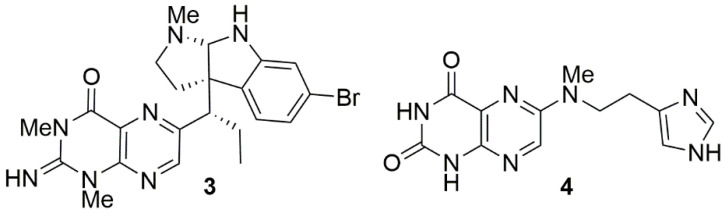
Marine pteridine natural products related to tedaniophorbasins A and B.

Comparison of the ^13^C chemical shift data for C-2, C-4, C-4a, C-5a, C-9a, C-10a, 1-*N*CH_3_, and 3-*N*CH_3_ observed for **1** with equivalent carbons reported for the pteridine metabolites urochordamine A (**3**) isolated from the ascidian *Ciona savignyi* [14] and asteropterin (**4**) isolated from the sponge *Asteropus simplex* [15] provided convincing evidence that **1** contained a pyrazine moiety, since all carbons apart from C-4a and C-10a were within 3.5 ppm of those reported in **3**, while C-2, C-4a, C-5a, and C-10a were within 3.1 ppm of those reported in **4** (Figure 4).

**Figure 5:**
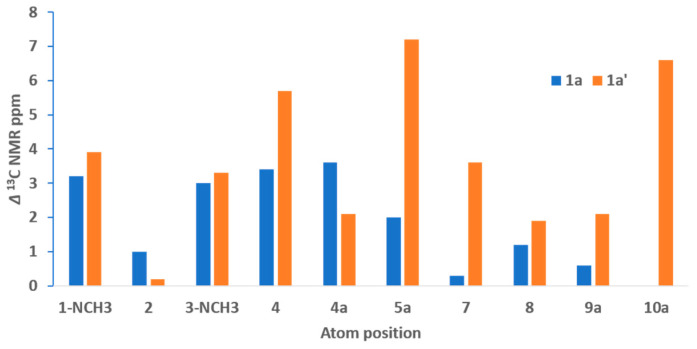
Calculated absolute ^13^C NMR chemical shift deviations for density functional theory (DFT)-calculated δ_C_ compared to experimental δ_C_ for the two alternative regioisomers **1a** and **1a′** of tedaniophorbasin A.

Unfortunately, the lack of pairs of protons proximal through space or protons within three bonds of more than one nitrogen atom meant that neither ROESY nor ^1^H/^15^N HMBC experiments would be useful to define the regiochemistry of the thiomorpholino moiety. Therefore, computational methods using density functional theory (DFT) GIAO-calculated ^13^C NMR chemical shifts were used to predict the most probable isomer for **1** (structures **1a** or **1a′**). The DFT-calculated ^13^C NMR isotropic shielding values were scaled to account for the heavy atom effect of sulfur [16] attached to C-8 and C-9a in **1a**, and C-5a and C-7 in **1a′**, and compared with tedaniophorbasin A’s (**1**) experimental ^13^C NMR data. The DFT-calculated ^13^C NMR data clearly indicated that structure **1a** (mean absolute error (MAE) = 1.8) containing the N and S atoms at positions 6 and 9, respectively, was a better match with **1′**s experimental ^13^C NMR data compared with that obtained for the alternate structure **1a′** (MAE = 3.7, see supplementary material). The DFT-calculated ^13^C NMR resonances in **1a′** with deviations >5.5 ppm were C-4, C-5a, and C-10a, whereas none of the calculated ^13^C NMR resonances in **1a** deviated from the observed data by more than 3.6 ppm (Figure 5). Tedaniophorbasin A therefore possesses a novel 2-imino-1,3-dimethyl-2,3,7,8-tetrahydro-1*H*-[1,4]thiazino [3,2-g]pteridin-4(6*H*)-one skeleton.

Tedaniophorbasin B (**2**) was isolated as a fluorescent yellow gum. A mass ion (MH^+^) at *m/z* 266.0706 (Δ 0 ppm) was consistent with **2** possessing a molecular formula of C_10_H_11_N_5_O_2_S. Its ^1^H and ^13^C NMR data (Table 1) were almost identical with that of **1**, with the most notable difference being the absence of the NH_2_ resonance at δ_H_ 9.05. The COSY (Figure S13) (6-NH/7-CH_2_, 7-CH_2_/8-CH_2_) and HMBC (Figures S15–S17) (1-*N*CH_3_/C-2, C-10a; 3-*N*CH_3_/C-2, C-4; 6-NH/C-8, C-9a; H-7/C-5a, C-8; and H-8/C-7, C-9a) correlation data for **2** was identical with that observed for **1**, indicating that the two molecules possessed the same molecular framework. Considering that the molecular formula for **2** differed from **1** by the replacement of a NH_2_^+^ with an oxygen, the most logical explanation is that **2** is the 2-oxo derivative of **1**. The structure proposed for tedaniophorbasin B (**2a**) is also supported by ^13^C DFT (GIAO) NMR calculations (MAE = 1.3, compared to MAE = 3.2 for the 6-S, 9-NH regioisomer (**2a′**) (see supplementary material)). Tedaniophorbasin B is therefore the 2-oxo derivative of **1** and contains a novel 2-oxo-1,3-dimethyl-2,3,7,8-tetrahydro-1*H*-[1,4]thiazino[3,2-g]pteridin-4(6*H*)-one skeleton.

**Figure 6:**
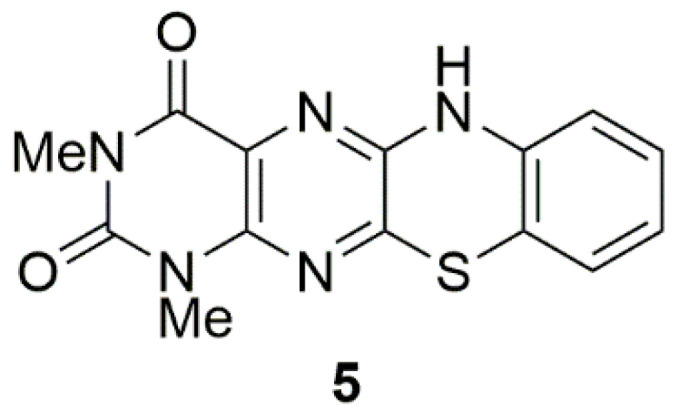
Most closely related synthetic pteridine derivative to the tedaniophorbasins.

The tedaniophorbasins are structurally unique compounds, possessing a ring system that has not been reported either naturally or synthetically. The closest related structure is the synthetic 7,8-benzo derivative (**5**) prepared through reaction of 6,7-dichloro-1,3-dimethyllumazine with 2-aminothiophenol (Figure 6) [17]. A similar reaction between mercaptoethylamine and 6,7-dichloro-1,3-dimethyllumazine could yield tedaniophorbasin B (**2**). Marine pteridine alkaloids have previously been isolated from polychaete worms [18], sponges [15], ascidians [14,19], and fungi [20]. All previously reported marine pteridine alkaloids, however, have been unsubstituted at C-7, thus making the tedaniophorbasins the first C-7 substituted pteridines to be reported from a marine source. There have not been any previous chemical investigation of sponges from the genus *Tedaniophorbas*.

Tedaniophorbasins A (**1**) and B (**2**) were tested for their ability to inhibit the growth of chloroquine-sensitive (3D7) and chloroquine-resistant (Dd2) strains of the malaria parasite *Plasmodium falciparum* [21], inhibition of the growth of the trypanosome *Trypanosoma brucei brucei* [22], and cytotoxicity towards pancreatic (Bx-PC-3, Panc-1, and Su-86-86) and breast cancer (BT-474, MCF-10A, and MDA-MB-231) cell lines [23] (assays that we routinely run in our labs), however, both compounds were inactive against all targets at the highest concentration (40 µM) tested.

The lack of cellular bioactivity prompted us to question the ecological role for these intensely fluorescent molecules, since the intensely fluorescent yellow color of the sponge can be attributed to the tedaniophorbasins. The fluorescence spectra for both **1** and **2** (Figure 2) showed significant Stokes shifts of 14,000 cm^−1^ (280 nm ex/490 nm em) and 3400 cm^−1^ (420 nm ex/490 nm em), suggesting excited state reactions and a change in dipole moments, respectively. The cyan fluorescence of tedaniophorbasin A (**1**) and B (**2**) further suggests that the compounds might act as donor luminophores for bioluminescence, while their UV absorbance between 280 and 419 nm suggests a role as sunscreens, since both compounds absorb strongly in the UVA (315–400 nm) and UVB (280–315 nm) wavelength bands.

## 3. Materials and Methods

### 3.1. General Chemistry Experimental Procedures

NMR spectra were recorded at 30 °C on a Varian Inova 600 MHz NMR spectrometer (Pola Alto, CA, USA) equipped with a cryoprobe. Samples were dissolved in DMSO-*d*_6_, and the solvent peak was used as the reference at the chemicals shifts δ_H_ 2.50 ppm and δ_C_ 39.52 ppm. LRESIMS data were recorded on a Water ZQ ESI mass spectrometer (Milford, MA, USA). (+)-High-resolution electrospray ionization mass spectrometry (HRESIMS) was used to determine the accurate molecular weight and molecular formula of the isolated compounds. HRESIMS were recorded on a Applied Biosystems Mariner Biospectrometry TOF workstation (Foster City, CA, USA) using positive electrospray ionization. Ultraviolet (UV) spectra were acquired on a Shimadzu UV-1800 UV spectrophotometer (Kyoto, Japan), and infrared (IR) measurements were recorded on a Bruker Tensor 27 spectrometer (Zürich, Switzerland). Fluorescence intensity spectra were recorded in MeOH by 3D top scanning on a Tecan Spark Plate Reader (Männedorf, Switzerland) with excitation wavelengths from 250 to 700 nm incremented by 10 nm and emissions intensity recorded from 280 to 700 nm at 10 nm steps at each excitation wavelength. HPLC separations were performed on a Merck Hitachi L-7100 pump equipped with a Hitachi L-7455 PDA detector, D-7000 interface (Tokyo, Japan), and a Gilson 215 liquid handler (Middleton, WI, USA) to collect the fractions. The guard column (20 mm × 10 mm) was packed with Altech Davisil 30–40 µm 60 Å C_18_ silica gel (Columbia, MD, USA). The guard column was attached before the preparative column, a Thermo Fisher Scientific BetaSil C_18_ (150 mm × 21.2 mm, 5 µm) (Waltham, MA, USA). The solvents used were HPLC grade MeOH (Lab-Scan) (Barcelona, Spain), Milli-Q PF (Sartorius, Göttingen, Germany) filtered water, and spectroscopy grade trifluoroacetic acid (TFA, Alfa Aesar) (Ward Hill, MA, USA).

### 3.2. Collections, Extraction, and Isolation

The sponge specimen *Tedaniophorbas ceratosis* (Ridley & Dendy, 1886) (Order: Poecilosclerida; Family: Acarnidae) (sp. number QM1179) was hand collected by SCUBA from Wommin Reef (10 m) just south of Cook Island, Northern NSW, Australia, in January 2010. A voucher specimen (QM G331842) is housed in the Queensland Museum. The specimen was identified by Dr. J. N. A. Hooper.

The sponge *Tedaniophorbas ceratosis* (20 g, wet weight) was chopped into small pieces and exhaustively extracted with MeOH (6 × 200 mL) in an ultrasonic bath (20 min. per extraction) to afford a yellow brown residue (0.892 g). This extract was then separated by reversed phase C_18_ HPLC gradient elution from H_2_O to MeOH (containing 1% TFA) over 60 min. and then eluted with MeOH (containing 1% TFA) for 10 min. to afford 70 fractions. Fractions 17 and 18 contained tedaniophorbasin A **1** and *N*-methyltryptamine, which were subsequently separated by recrystallization from MeOH. Tedaniophorbasin A (**1**) was crystallized from the solution to leave *N*-methyltryptamine in the supernatant. Fraction 20 eluting with 66% H_2_O/34% MeOH yielded pure *N*-methyltryptamine, and fraction 27 eluting with 55% H_2_O/45% MeOH (all containing 1% TFA) yielded pure tedaniophorbasin B (**2**).

Tedaniophorbasin A (**1**): yellow crystals (18.5 mg, 0.09%); m.p. 316 °C; UV (MeOH) λ_max_ (log ε) 223 (4.12), 280 (4.02), 296 (3.88), 419 (3.87) nm; IR υ_max_ (film) 3325, 3058, 2966, 2929, 1712, 1679, 1649, 1567, 1557, 1202, 1132 cm^−1^; FLR (MeOH) λ_ex_ 280, λ_em_ 490 nm, λ_ex_ 419, λ_em_ 490 nm; ^1^H and ^13^C NMR data, Table 1; (+) HRESIMS *m/z* 265.0863 (calcd for C_10_H_13_N_6_OS, 265.0866).

Tedaniophorbasin B (**2**): yellow oil (1.3 mg, 0.007%); UV (MeOH) λ_max_ (log ε) 219 (4.01), 277 (3.77), 302 (3.57), 416 (3.55) nm; IR υ_max_ (film) 3302, 2918, 2850, 1679, 1200, 1135 cm^−1^; FLR (MeOH) λ_ex_ 277, λ_em_ 490 nm, λ_ex_ 416, λ_em_ 490 nm; ^1^H and ^13^C NMR data, Table 1; (+) HRESIMS *m/z* 266.0706 (calcd for C_10_H_12_N_5_O_2_S, 266.0706).

### 3.3. DFT ^13^C NMR Calculations

A thorough conformer search was performed on **1a**, **1a′**, **2a**, and **2a′** using MCMM with the OPLS3 forcefield within the SchrÖdinger Macromodel 2016 software suite operating on Windows 10. All conformers >21.0 kJ/mol from the energy minimum were discarded. The resultant conformational suites underwent DFT geometry optimization in the gas phase using the B3LYP/6-31G(d) functional and basis set in Gaussian 16 [24]. Modified python scripts based on the Willoughby protocol [25] were used to confirm that the geometry-optimized conformers were true energy minima and to confirm the absence of negative vibrational frequencies. The DFT (GIAO) ^13^C NMR shielding values were calculated on the geometry-optimized structures using the ωB97xD/6-31G(d) level of theory. The subsequent DFT isotropic shielding values were then Boltzmann averaged across each of the conformational suites [25] and scaled (with carbons attached to sulfur adjusted binomially) using the procedure reported by Kutateladze et al. [16]. Finally, the scaled ^13^C NMR chemical shifts for the structural isomers (**1a** and **1a′**; **2** and **2a′**) were analyzed using the sDP4+ method [26], however, the ωB97xD/6-31G(d) functional is not inherent to the Grimblat method, therefore a small amount of caution should be applied to this result (see supplementary material).

### 3.4. Biological Activity Testing

Biological profiling of compounds was undertaken using well-established protocols. Antiplasmodial activity was determined by testing compounds against chloroquine-sensitive (3D7) and chloroquine-resistant (Dd2) strains of *Plasmodium falciparum* as previously comprehensively described by Duffy and Avery [21].

Antitrypanosomal activity of compounds against *Trypanosoma brucei brucei* was undertaken as previously described by Sykes and Avery [22].

Evaluation of compounds for anti-proliferative activity against the breast cancer cell lines BT-474, MCF-10A, and MDA-MB-231 and pancreatic cell lines Bx-PC-3, Panc-1, and Su-86-86 was undertaken as described in detail by Lovitt et al. [23].

## 4. Conclusions

Analysis of a sponge *Tedaniophorbas ceratosis* collected from the Australian south Pacific coast led to the isolation of two highly fluorescent alkaloids, tedaniophorbasins A and B. Both possess skeletons that are novel. Tedaniophorbasin A possesses a novel 2-imino-1,3-dimethyl-2,3,7,8-tetrahydro-1*H*-[1,4]thiazino[3,2-g]pteridin-4(6*H*)-one skeleton, while tedaniophorbasin B is the 2-oxo derivative of tedaniophorbasin A. Their structures were supported by comparison of experimental and DTF-calculated ^13^C NMR data. Both compounds were inactive at 40 µM when tested for antiplasmodial, anticancer, and antitrypanosomal activity. This study highlights the fact that the south Pacific region contains marine genera that, to date, have not been investigated, and that these marine organisms hold the potential to contain novel chemistry. Futures studies of south Pacific marine organisms will undoubtedly lead to further discoveries of novel chemistry.

## Figures and Tables

**Table 1:** NMR data for tedaniophorbasins A and B (**1** and **2**) recorded in DMSO *d*_6_ at 30 °C ^a^.

	1		2	
Position	δc, Type	δ_H_, Mult. (*J* in Hz)	δc, Type	δ_H_, Mult. (*J* in Hz)
1-*N*CH_3_	31.3, CH_3_	3.66, s	28.9, CH_3_	3.43, s
2	151.0, C	-	150.0, C	-
2-NH_2_^+^	-	9.05, bs	-	-
3-*N*CH_3_	30.3, CH_3_	3.46, s	28.2, CH_3_	3.27, s
4	157.4, C	-	159.9, C	-
4a	120.6, C	-	123.6, C	-
5a	148.0 ^b^, C	-	146.6, C	-
6	-	8.15, t (2.7)	-	7.64, t (2.7)
7	40.6, CH_2_	3.65, m	40.7, CH_2_	3.61, m
8	26.3, CH_2_	3.34, m	26.5, CH_2_	3.29, m
9a	147.9 ^b^, C	-	146.0, C	-
10a	137.6, C	-	139.8, C	-

^a^ Spectra recorded at 600 MHz for ^1^H and 150 MHz for ^13^C; ^b^ assignments are interchangeable.

## Data Availability

The data presented in this study are available in the supplementary Materials file associate with this article.

## Supplementary Materials

The following are available online at https://www.mdpi.com/1660-3397/19/2/95/s1: Figures S1–S8: ^1^H NMR, ^13^C NMR, COSY, HSQC, HMBC spectra of tedaniophorbasin A (**1**); Figures S10–S17: ^1^H NMR, ^13^C NMR, COSY, HSQC, HMBC spectra of tedaniophorbasin B (**2**). HRESIMS spectra for tedaniophorbasin A (**1**): Figure S9 and tedaniophorbasin B (**2**): Figure S18, Tables S1–S4 Calculated DFT (GIAO) ^13^C NMR Chemical Shift for **1a**, **1a′**, **2a**, and **2a′** compared to experimental ^13^C NMR data.

## References

[1] Feher M., Schmidt J.M. (2003). Property distributions: Differences between drugs, natural products, and molecules from combinatorial chemistry. J. Chem. Inf. Comput. Sci..

[2] Blunt J.W., Carroll A.R., Copp B.R., Davis R.A., Keyzers R.A., Prinsep M.R. (2018). Marine Natural Products. Nat. Prod. Rep..

[3] Pye C.R., Bertinb M.J., Lokeya R.S., Gerwick W.H., Linington R.G. (2017). Retrospective analysis of natural products provides insights for future discovery trends. Proc. Natl. Acad. Sci. USA.

[4] Henkel T., Brunne R.M., Müller H., Reichel F. (1999). Statistical Investigation into the Structural Complementarity of Natural Products and Synthetic Compounds. Angew. Chem. Int. Ed..

[5] Butler A.J., Rees T., Beesley P., Bax N.J. (2010). Marine Biodiversity in the Australian Region. PLoS ONE.

[6] Carroll A.R., Copp B.R., Davis R.A., Keyzers R.A., Prinsep M.R. (2019). Marine Natural Products. Nat. Prod. Rep..

[7] Carroll A.R., Wild S.J., Duffy S., Avery V.M. (2012). Kororamide A, a new tribrominated indole alkaloid from the Australian bryozoan *Amathia tortuosa*. Tetrahedron Lett..

[8] Kleks G., Duffy S., Lucantoni L., Avery V.M., Carroll A.R. (2020). Orthoscuticellines A–E, β-Carboline Alkaloids from the Bryozoan *Orthoscuticella ventricosa* Collected in Australia. J. Nat. Prod..

[9] Jennings L.K., Robertson L.P., Rudolph K.E., Munn A.L., Carroll A.R. (2019). Anti-prion Butenolides and Diphenylpropanones from the Australian Ascidian *Polycarpa procera*. J. Nat. Prod..

[10] Kleks G., Holland D.C., Kennedy E.K., Avery V.M., Carroll A.R. (2020). Antiplasmodial Alkaloids from the Australian Bryozoan *Amathia lamourouxi*. J. Nat. Prod..

[11] Jennings L.J., Prebble D.W., Xu M., Ekins M.G., Munn A.L., Mellick G.D., Carroll A.R. (2020). Anti-prion and α-Synuclein Aggregation Inhibitory Sterols from the Sponge *Lamellodysidea* cf. chlorea. J. Nat. Prod..

[12] Hayton J.H., Grant G.D., Carroll A.R. (2019). Three New Spongian Diterpenes from the Marine Sponge *Dendrilla rosea*. Aust. J. Chem..

[13] Pretsch E., Bühlmann P., Badertscher M. (2009). Structure Determination of Organic Compounds.

[14] Tsukamoto S., Hirota H., Kato H., Fusetani N. (1993). Urochordamines A and B: Larval Settlement/Metamorphosis-Promoting, Pteridine-Containing Physostigmine Alkaloids from the Tunicate *Ciona savignyi*. Tetrahedron Lett..

[15] Murayama S., Nakao Y., Matsunaga S. (2008). Asteropterin, an inhibitor of cathepsin B, from the marine sponge *Asteropus simplex*. Tetrahedron Lett..

[16] Kutateladze A.G., Reddy D.S. (2017). High-Throughput in Silico Structure Validation and Revision of Halogenated Natural Products Is Enabled by Parametric Corrections to DFT-Computed ^13^C NMR Chemical Shifts and Spin-Spin Coupling Constants. J. Org. Chem..

[17] Abou-Hadeed K., Pfleiderer W. (1996). Pteridines eVIII Reactions of 6, 7-Dichloro-I, 3-dimethyllumazine with Sulfur-Nucleophiles. Pteridines.

[18] Kakoi H., Tanino H., Okada K., Inoue S. (1995). 6-Acyllumazines from the marine polychaete, *Odontosyllis undecimdonta*. Heterocycles.

[19] Rudolph K.E., Liberio M.S., Davis R.A., Carroll A.R. (2013). Pteridine-, thymidine-, choline-and imidazole-derived alkaloids from the Australian ascidian, *Leptoclinides durus*. Org. Biomol. Chem..

[20] You M., Liao L., Hong S.H., Park W., Kwon D.I., Lee J., Noh M., Oh D.-C., Oh K.-B., Shin J. (2015). Lumazine Peptides from the Marine-Derived Fungus *Aspergillus terreus*. Mar. Drugs.

[21] Duffy S., Avery V.M. (2012). Development and optimization of a novel 384-well anti-malarial imaging assay validated for high-throughput screening. Am. J. Trop. Med. Hyg..

[22] Sykes M.L., Avery V.M. (2009). Development of an Alamar Blue™ Viability Assay in 384-Well Format for High Throughput Whole Cell Screening of *Trypanosoma brucei brucei* Bloodstream Form Strain 427. Am. J. Trop. Med. Hyg..

[23] Lovitt C.J., Shelper T.B., Avery V.M. (2013). Miniaturized three-dimensional cancer model for drug evaluation. Assay Drug Dev. Technol..

[24] Frisch M.J., Trucks G.W., Schlegel H.B., Scuseria G.E., Robb M.A., Cheeseman J.R., Scalmani G., Barone V., Petersson G.A., Nakatsuji H. (2016). Gaussian 16.

[25] Willoughby P.H., Jansma M.J., Hoye T.R. (2014). A Guide to Small-Molecule Structure Assignment through Computation of (^1^H and ^13^C) NMR Chemical Shifts. Nat. Protoc..

[26] Grimblat N., Zanardi M.M., Sarotti A.M. (2015). Beyond DP4: An Improved Probability for the Stereochemical Assignment of Isomeric Compounds Using Quantum Chemical Calculations of NMR Shifts. J. Org. Chem..

