# Supplementary material for: Tedaniophorbasins A and B—Novel Fluorescent Pteridine Alkaloids Incorporating a Thiomorpholine from the Sponge Tedaniophorbas ceratosis
Source: Mar Drugs. 2021 Feb 7;19(2):95. doi: 10.3390/md19020095 (PMC7915533; doi:10.3390/md19020095)
Supplement: Supplementary file 1 [file marinedrugs-19-00095-s001.pdf]

Supplementary Material

Tedaniophorbasins A and B, novel fluorescent pteridine alkaloids incorporating a thiomorpholine from the sponge *Tedaniophorbas ceratosis*

Asadhawut Hiranrat[1,2,3], Darren C. Holland[1,4], Wilawan Mahabusarakam [2,3], John N. A. Hooper[4,5], Vicky M. Avery[4,6], Anthony R. Carroll[1,4*]

[1]School of Environment and Science, Griffith University, Gold Coast, QLD 4222, Australia
[2]Department of Chemistry, Faculty of Science, Prince of Songkla University, Hat Yai, Songkhla 90112, Thailand.
[3]Natural Products Research Center, Faculty of Science, Prince of Songkla University, Hat Yai, Songkhla 90112, Thailand.
[4]Griffith Institute for Drug Discovery Institute, Griffith University, Brisbane, QLD 4111, Australia
[5]Queensland Centre for Biodiversity, Queensland Museum, South Brisbane, Queensland 4101, Australia
[6] Discovery Biology, Griffith University, Nathan, QLD 4111, Australia

∗ Corresponding author. Tel.: +6-175-552-9187; fax: +6-175-552-9047; e-mail: a.carroll@griffith.edu.au

Contents

**1)**	**NMR Spectra for Tedaniophorbasin A (1)**	**S2**
**2)**	**HRESIMS for Tedaniophorbasin A (1)**	**S10**
**3)**	**NMR Spectra for Tedaniophorbasin B (2)**	**S11**
**4)**	**HRESIMS for Tedaniophorbasin B (2)**	**S19**
**5)**	**Calculated DFT (GIAO) ^{13}C NMR Chemical Shifts for 1, 1a′, 2 and 2a′**	**S20**

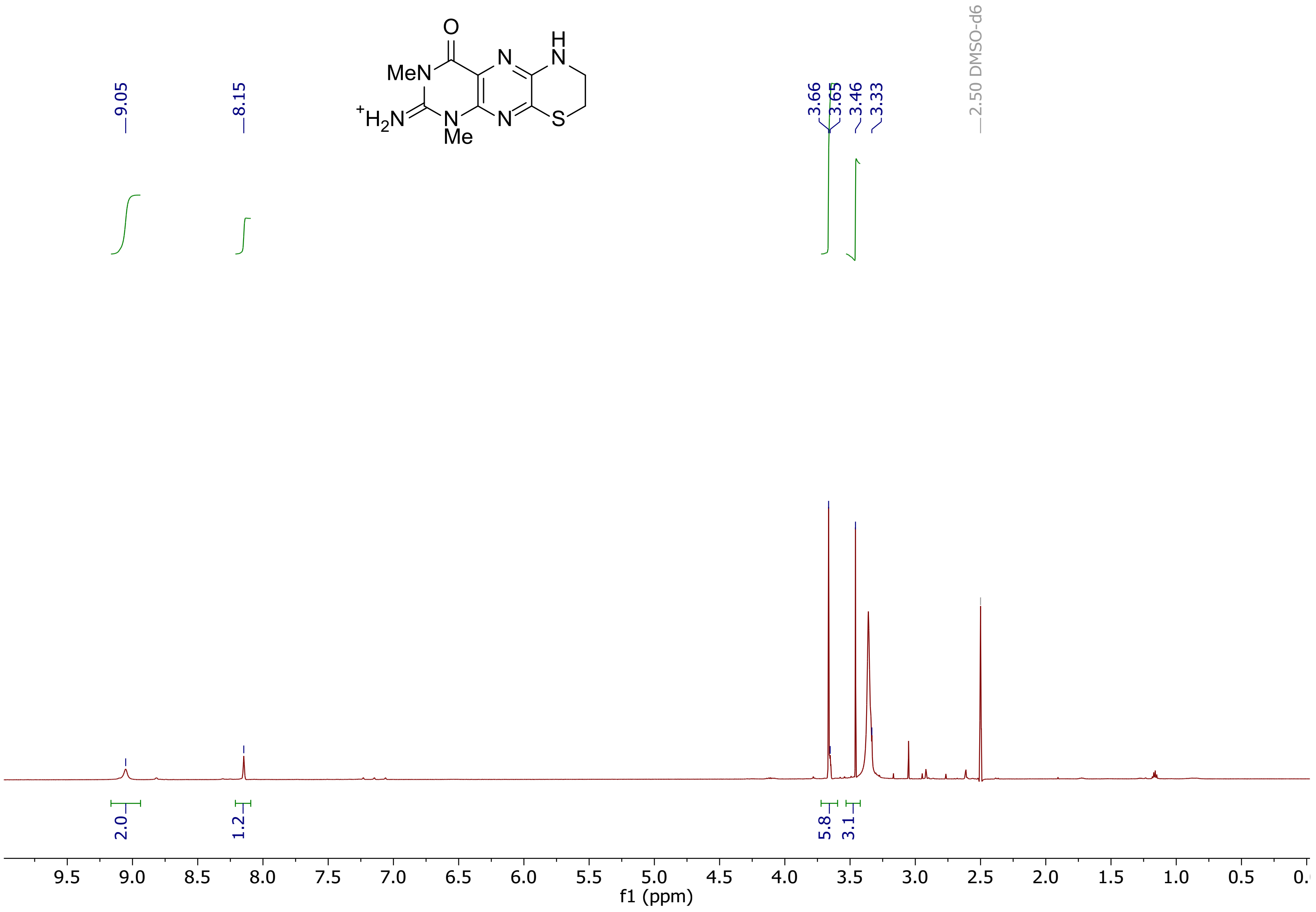

Figure S1: ^{1}H NMR spectrum (600 MHz) of tedaniophorbasin A (**1**) in DMSO-d_6.

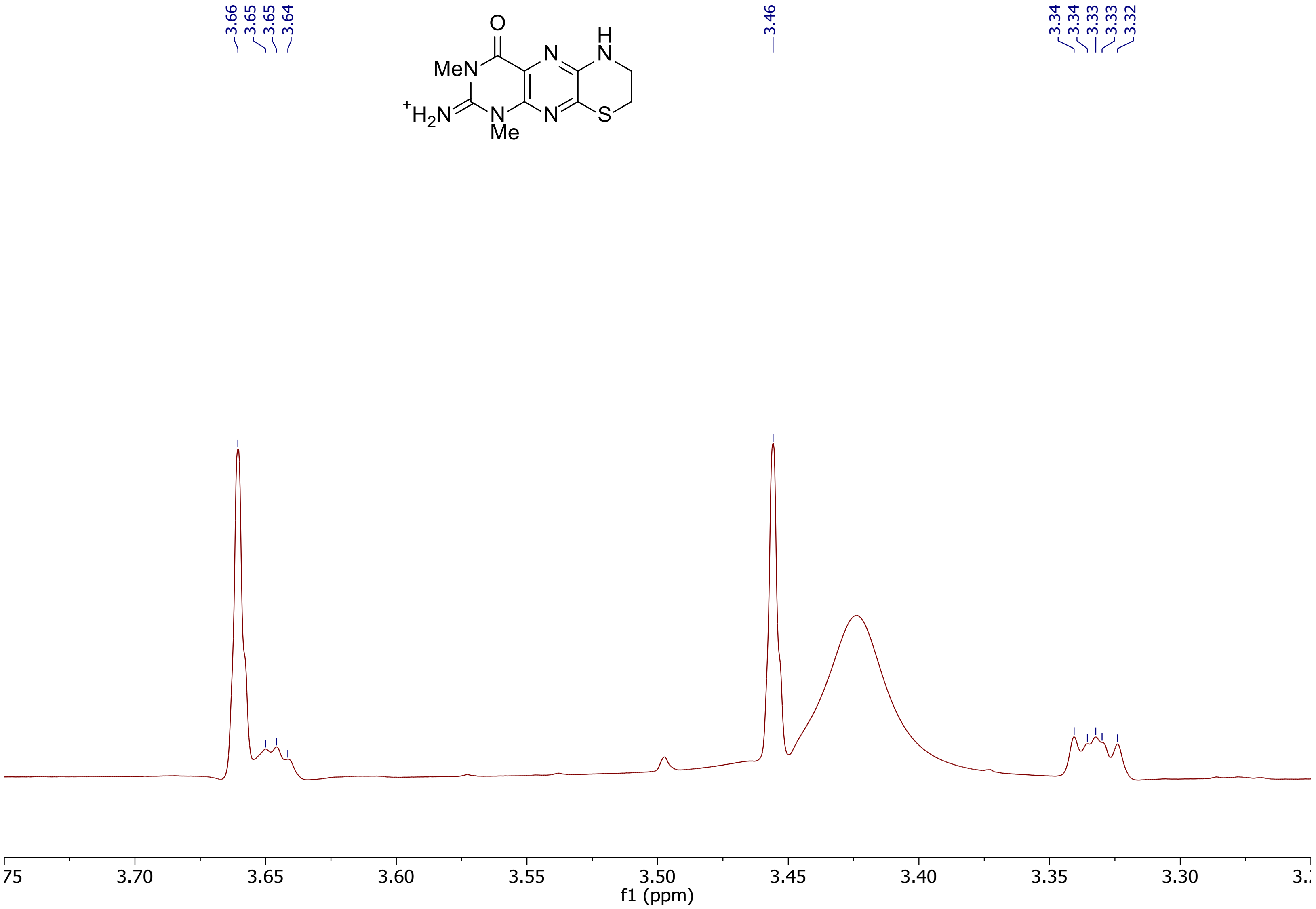

Figure S2: ^{1}H NMR spectrum (600 MHz) expansion of tedaniophorbasin A (**1**) in DMSO-d_6.

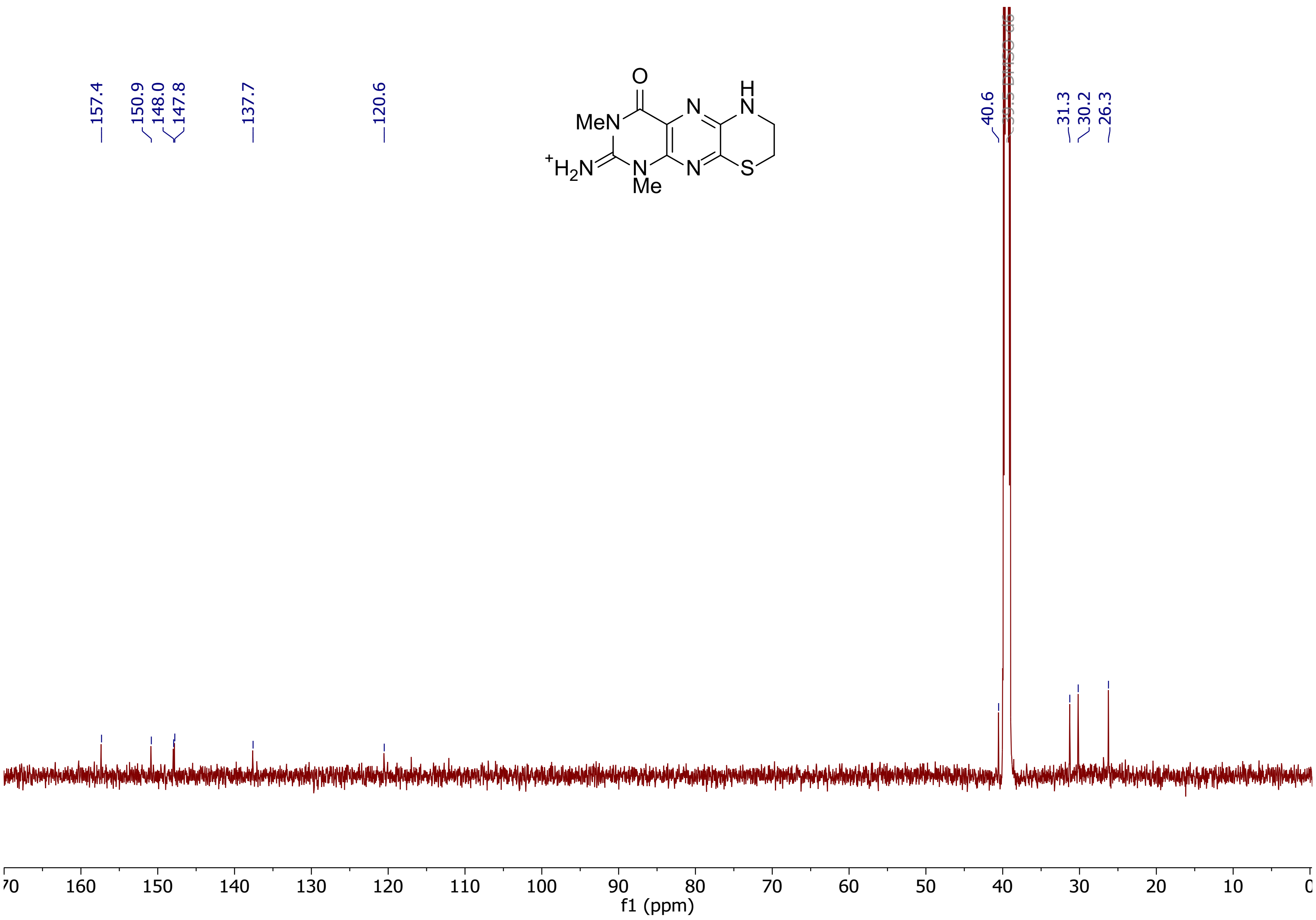

Figure S3: ^{13}C NMR spectrum (150 MHz) of tedaniophorbasin A (**1**) in DMSO-d_6.

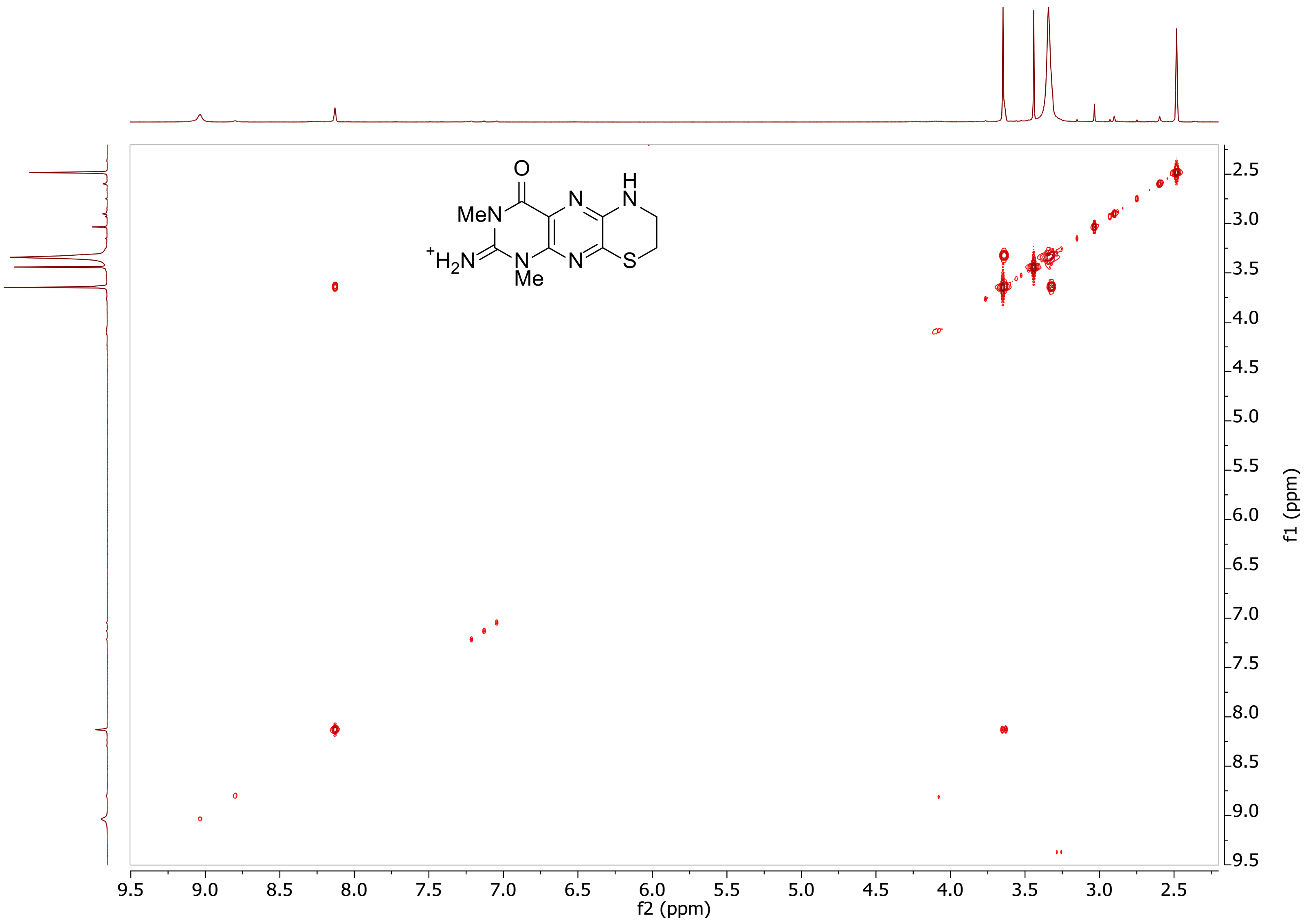

Figure S4: COSY NMR spectrum (600 MHz) of tedaniophorbasin A (**1**) in DMSO-d_6.

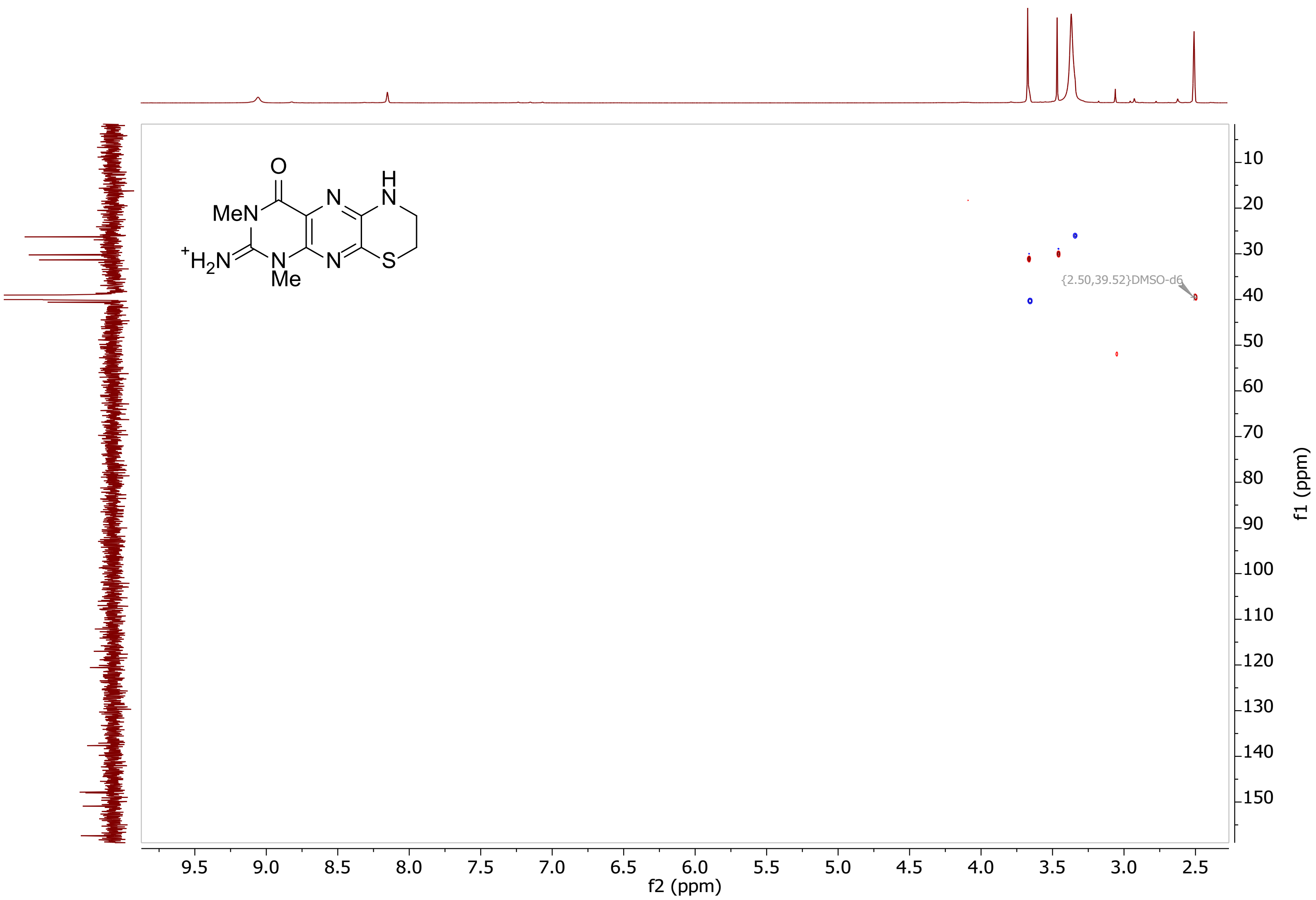

Figure S5: HSQC NMR spectrum (600 MHz) of tedaniophorbasin A (**1**) in DMSO-d_6.

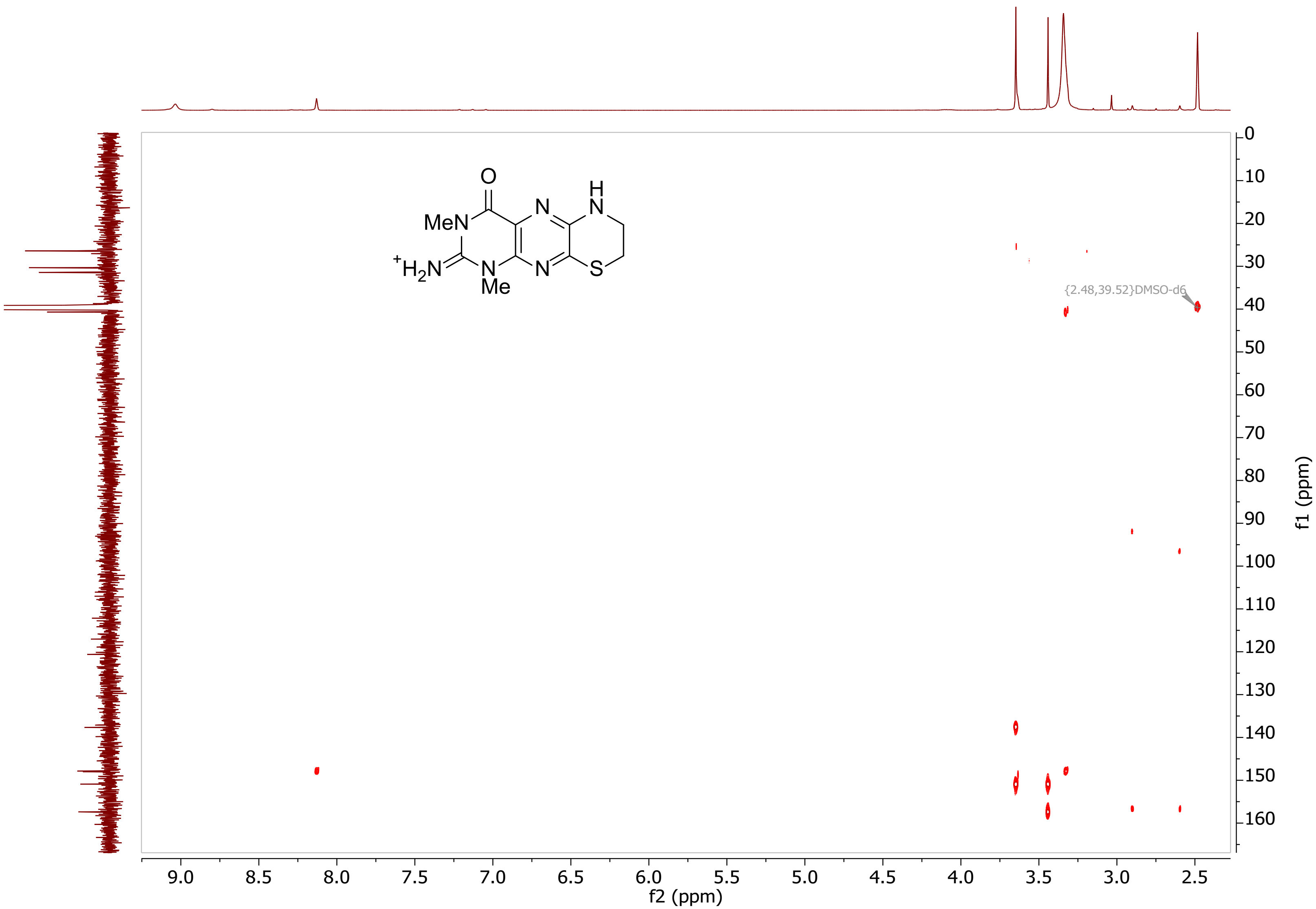

Figure S6: HMBC NMR spectrum (600 MHz) of tedaniophorbasin A (**1**) in DMSO-d_6.

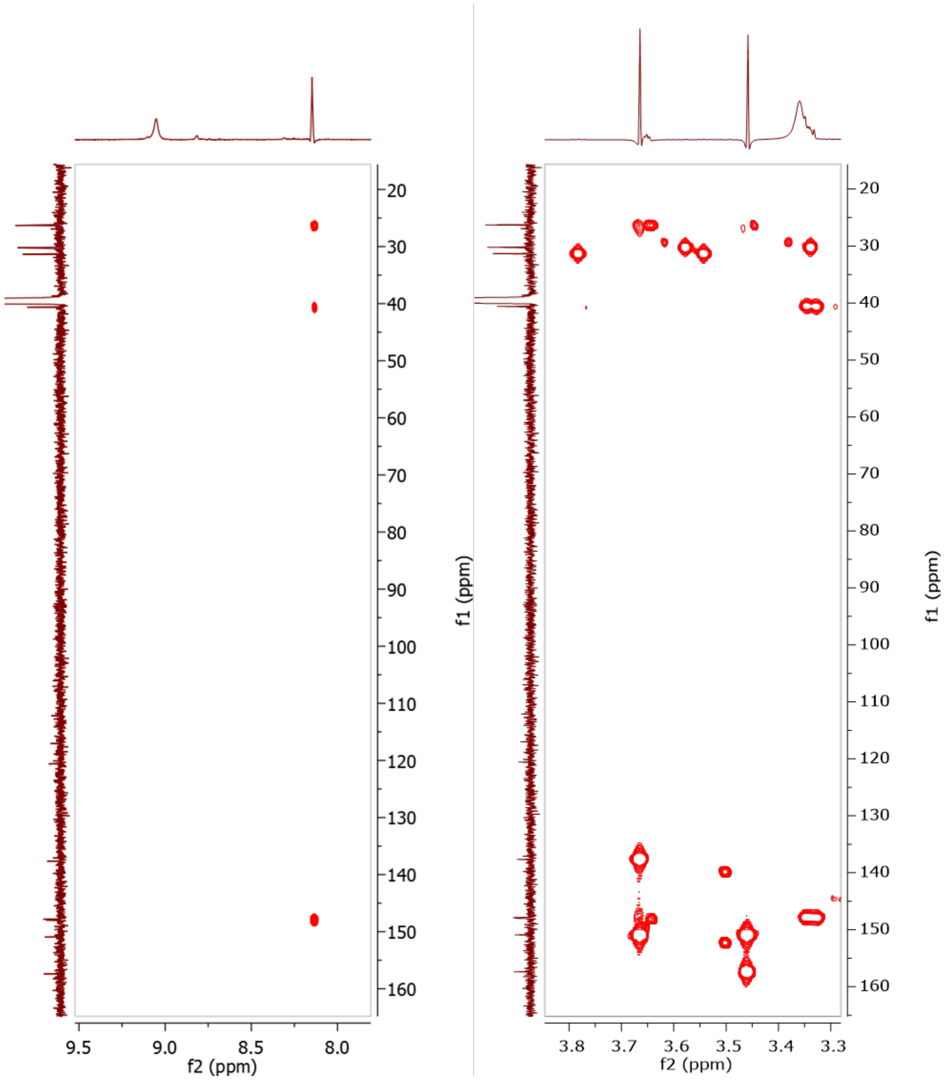

Figure S7: HMBC NMR spectrum (600 MHz) Expansions for tedaniophorbasin A (**1**) in DMSO-d_6.

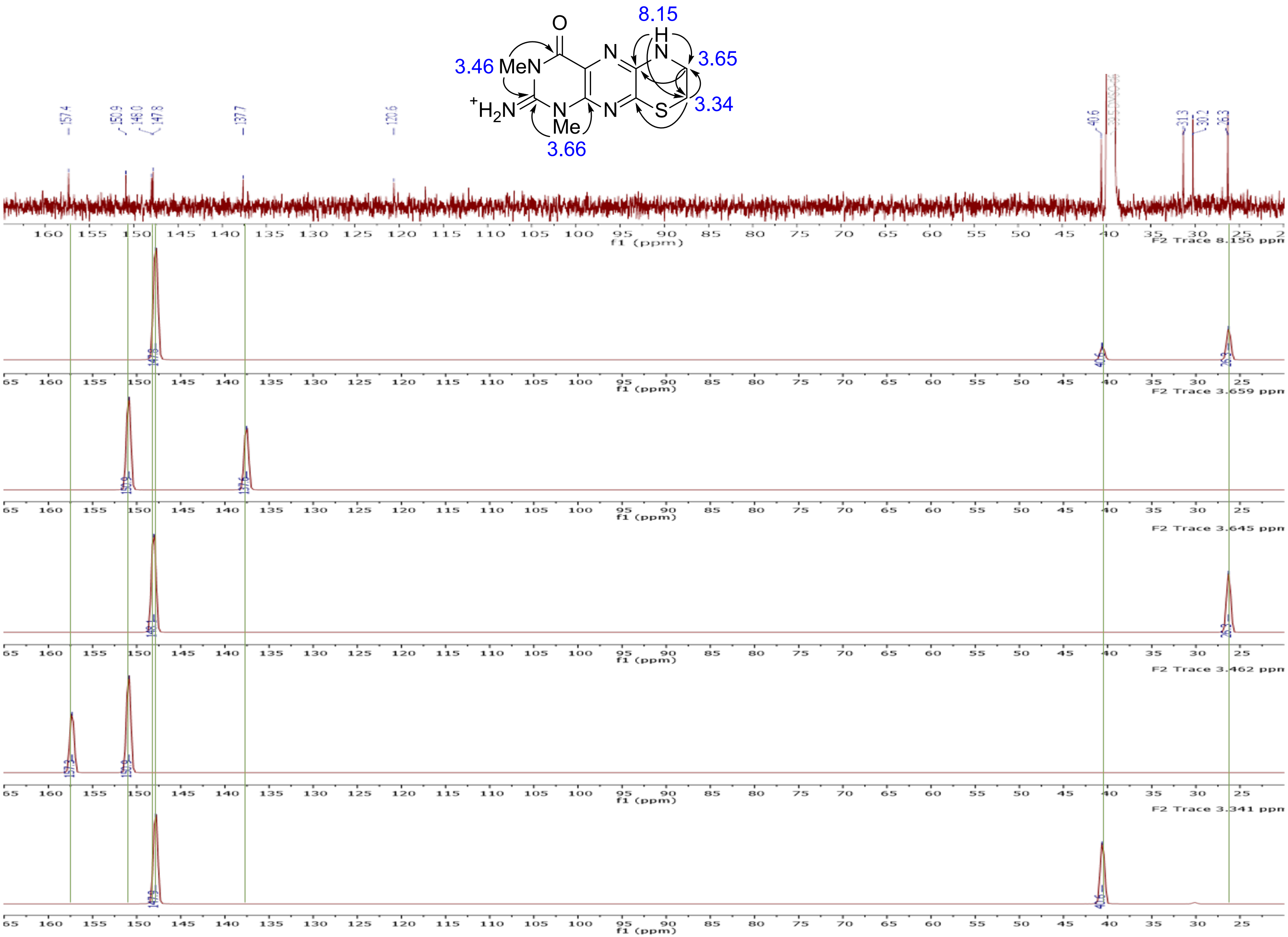

Figure S8: HMBC NMR Traces at each ^{1}H resonance chemical shift (600 MHz) of tedaniophorbasin A (**1**) in DMSO-d_6.

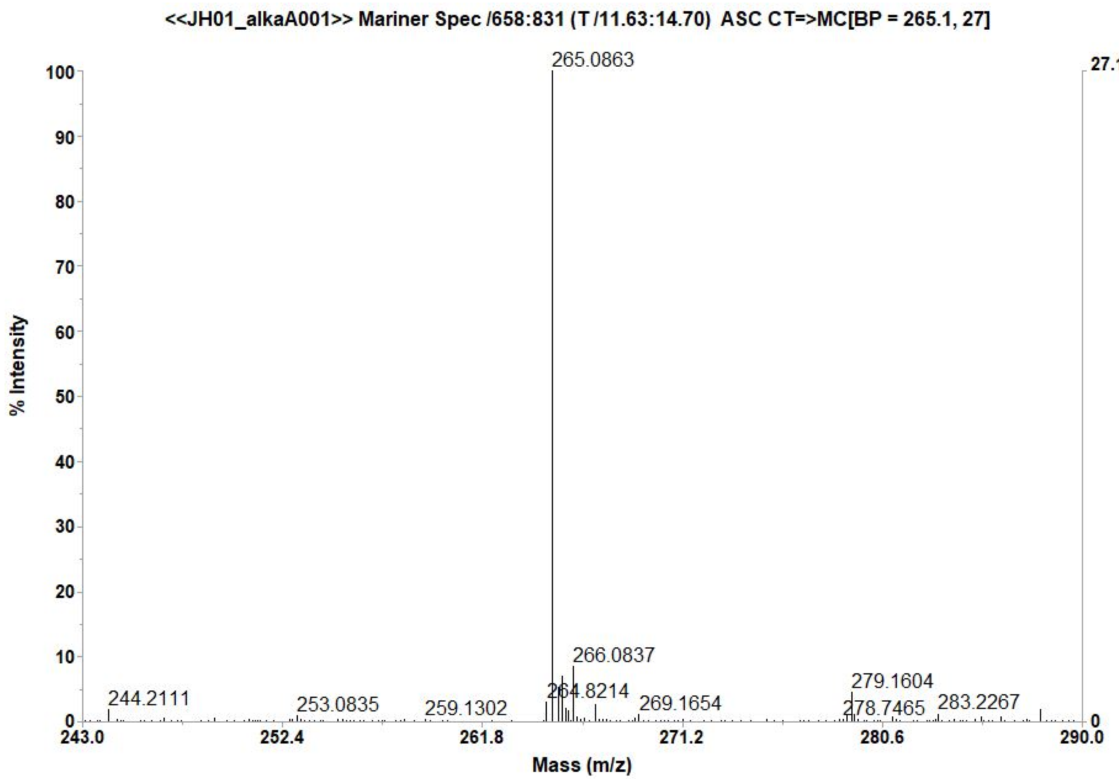

Figure S9: HRESIMS $[M+H]^+$ of tedaniophorbasin A (**1**)

#	MF	Monoisotopic mass	Ionization	*m/z*	PPM	mDa	unsaturation
1	$C_2H_9N_{12}O_4$	265.0870	H^+	265.0864	-0.46	-0.12	4.5
2	$C_{10}H_{21}N_2S_3$	265.0867	H^+	265.0861	0.61	0.16	1.5
3	$C_{10}H_{13}N_6OS$	265.0872	H^+	265.0866	-1.16	-0.31	7.5
4	$C_{17}H_{13}O_3$	265.0865	H^+	265.0859	1.43	0.38	11.5
5	$C_9H_{17}N_2O_5S$	265.0858	H^+	265.0853	3.89	1.03	2.5

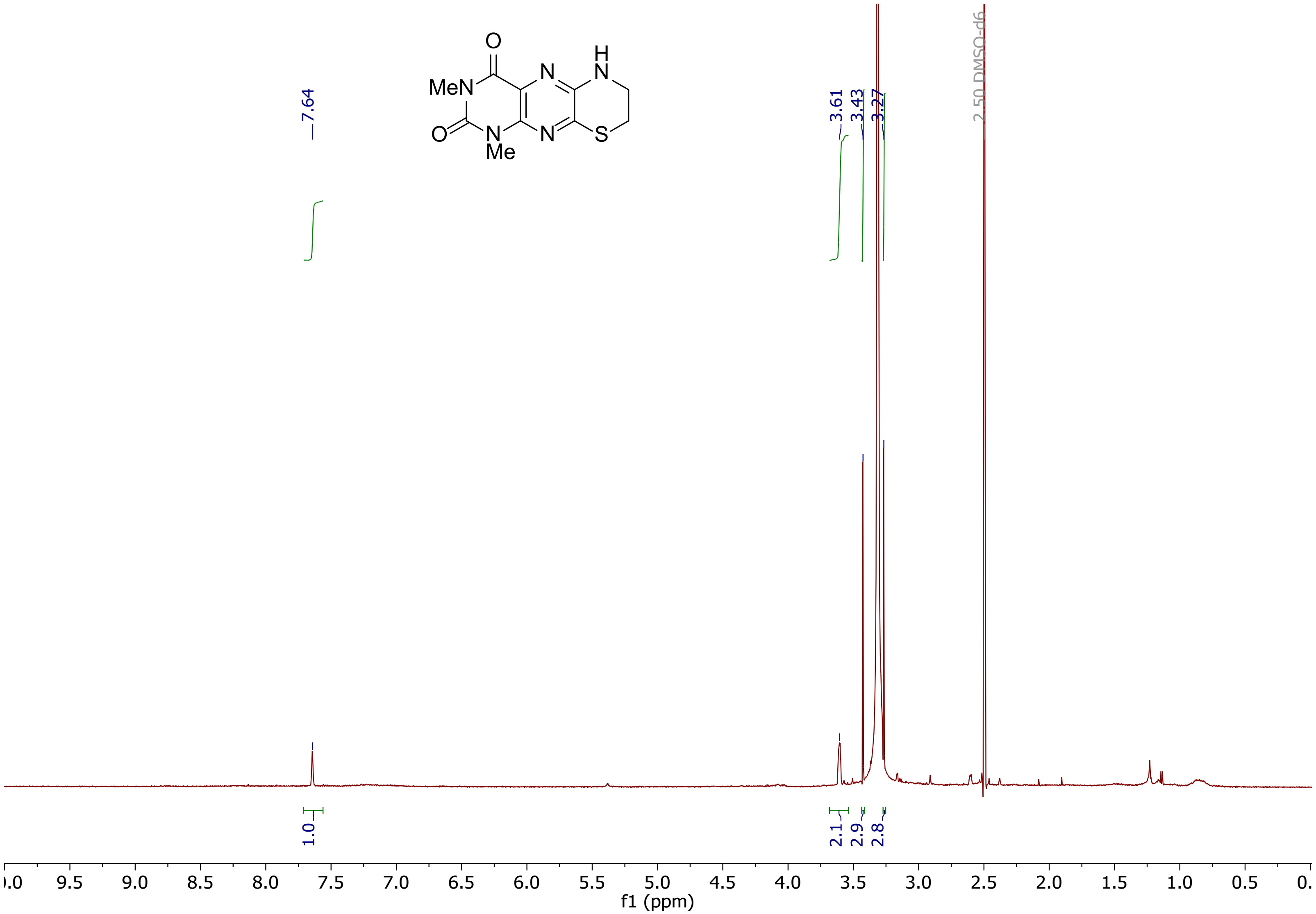

Figure S10: ^{1}H NMR spectrum (600 MHz) of tedaniophorbasin B (**2**) in DMSO-d_6.

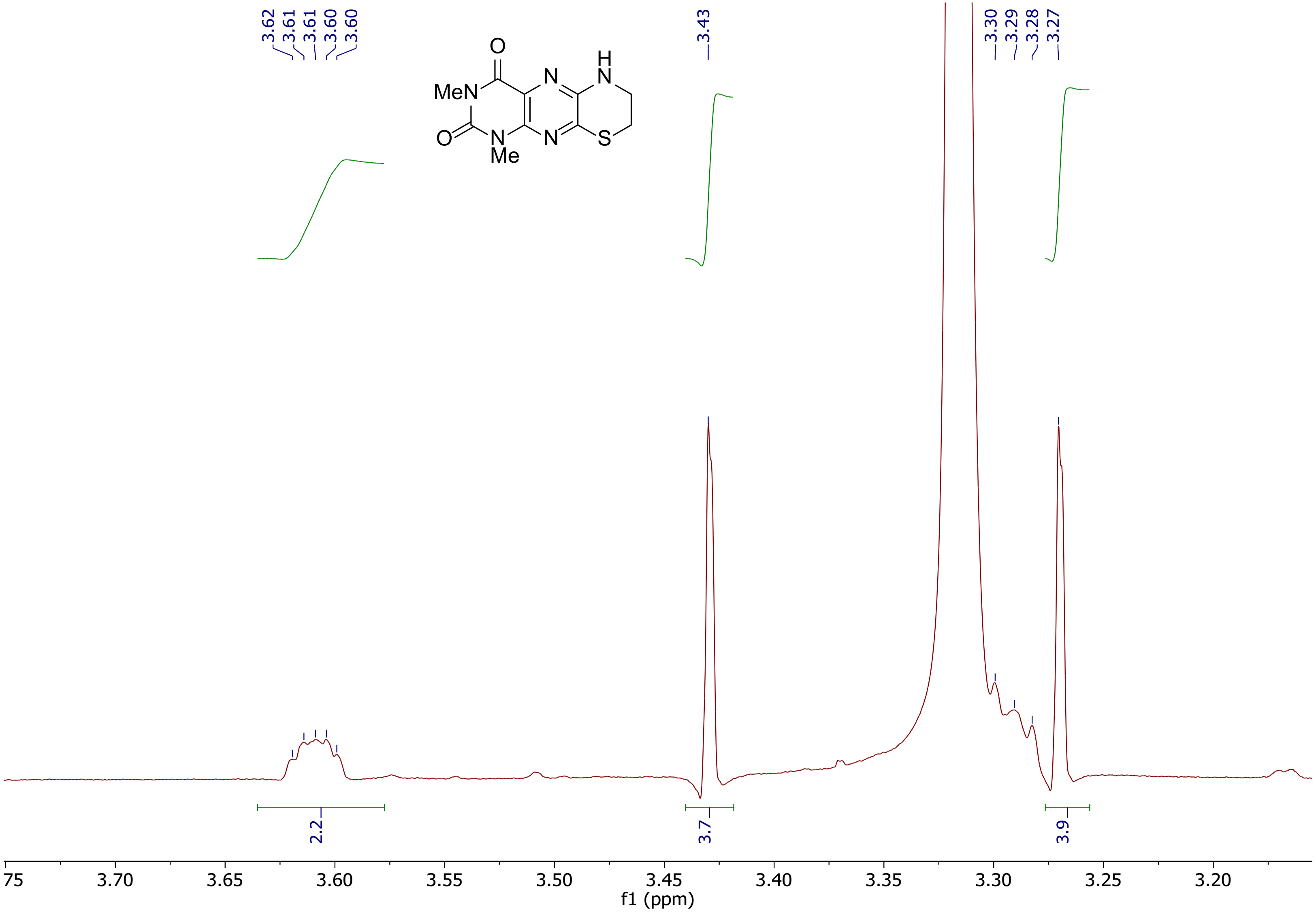

Figure S11: ^{1}H NMR spectrum (600 MHz) expansion of tedaniophorbasin B (**2**) in DMSO-d_6.

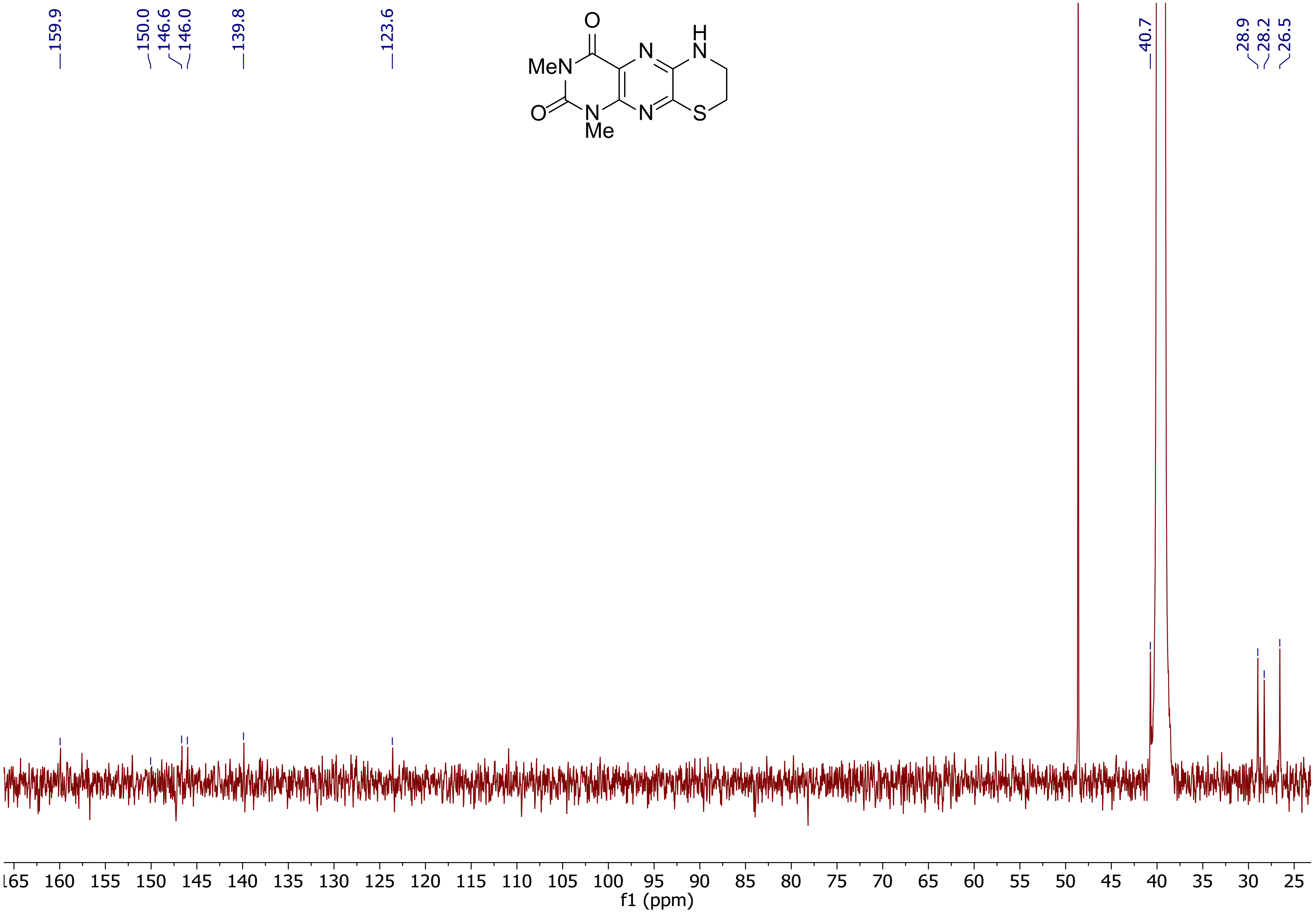

Figure S12: ^{13}C NMR spectrum (150 MHz) of tedaniophorbasin B (**2**) in DMSO-d_6.

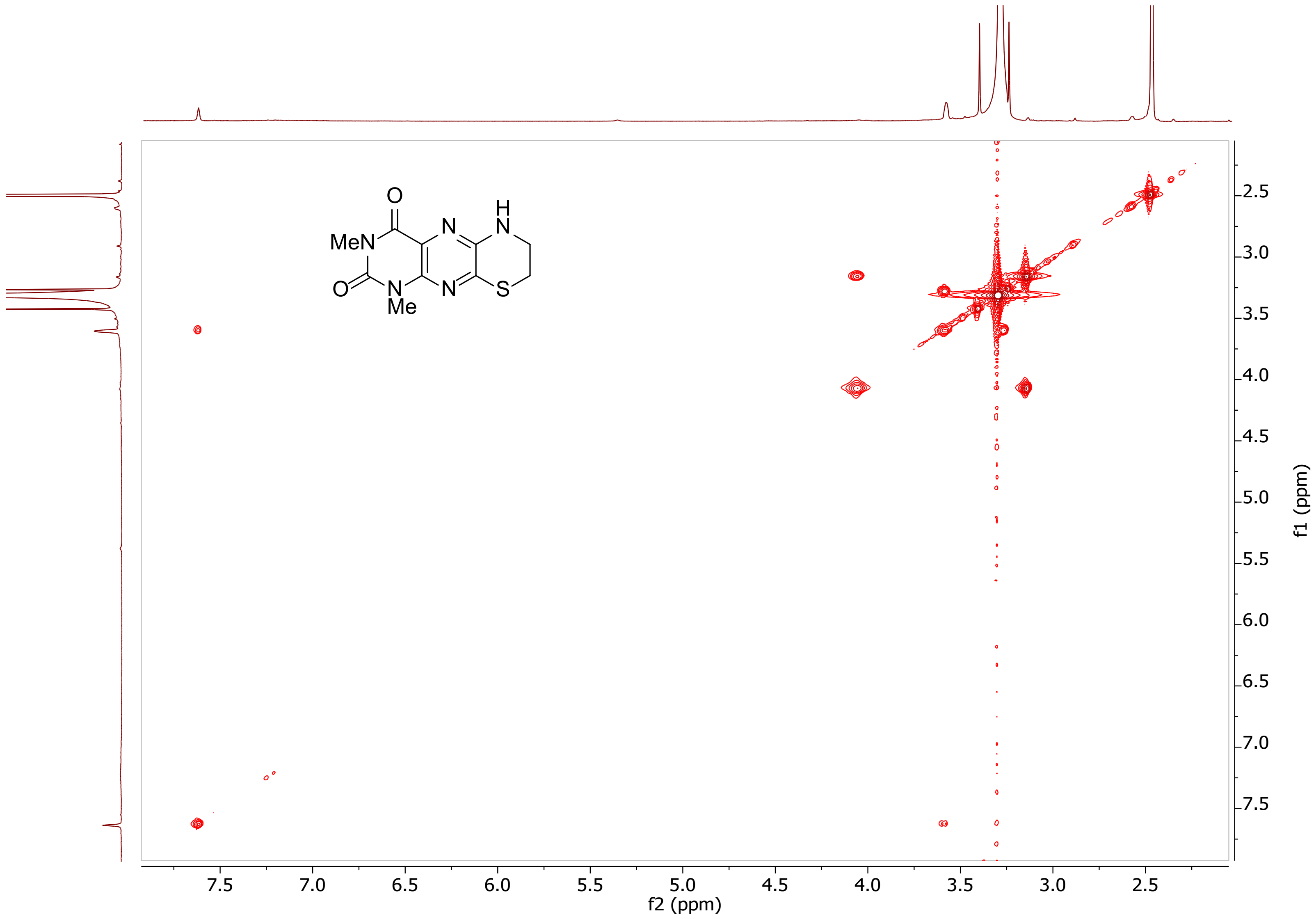

Figure S13: COSY NMR spectrum (600 MHz) of tedaniophorbasin B (**2**) in DMSO-d_6.

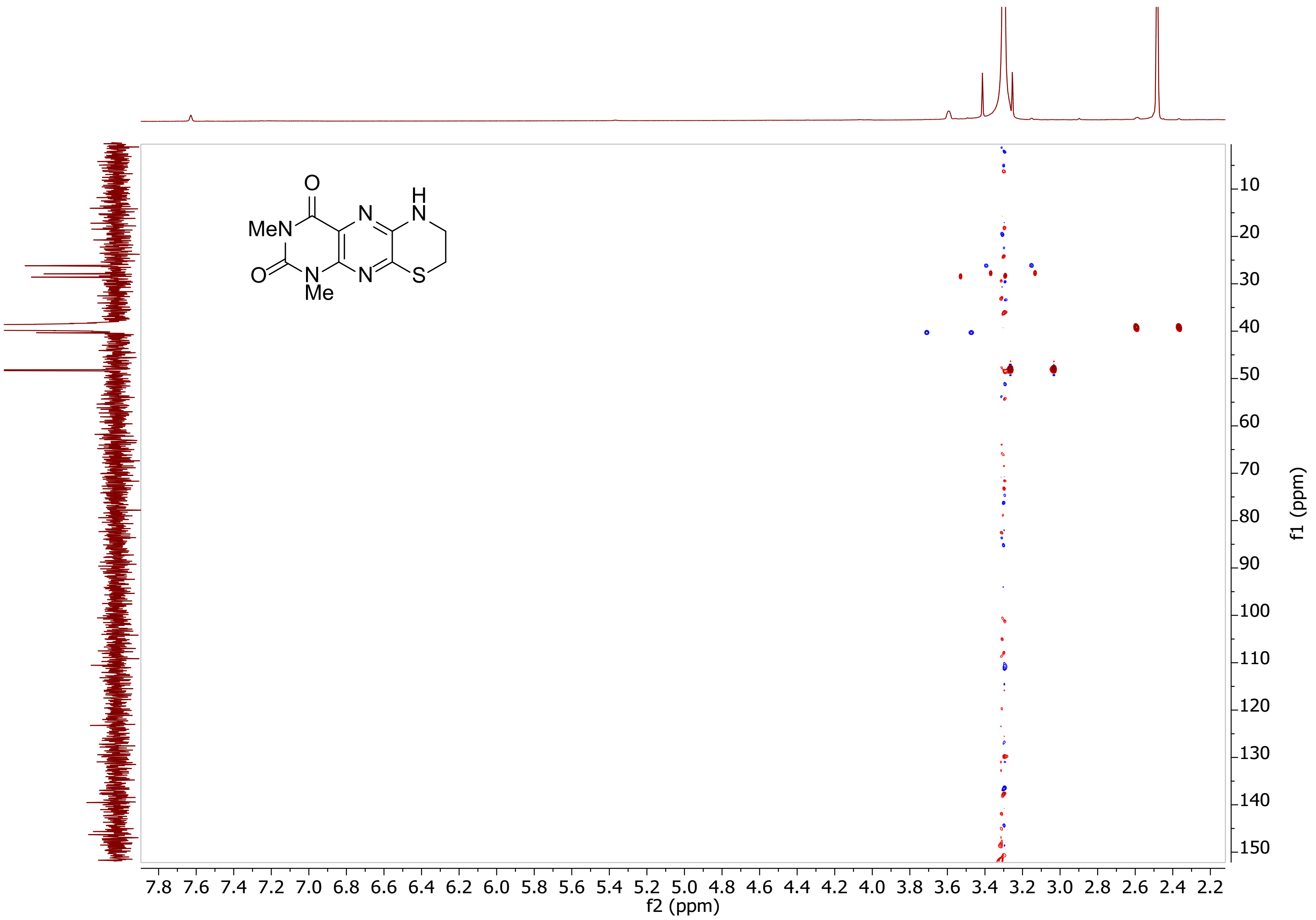

Figure S14: Coupled Edited HSQC NMR spectrum (600 MHz) of tedaniophorbasin B (**2**) in DMSO-d_6.

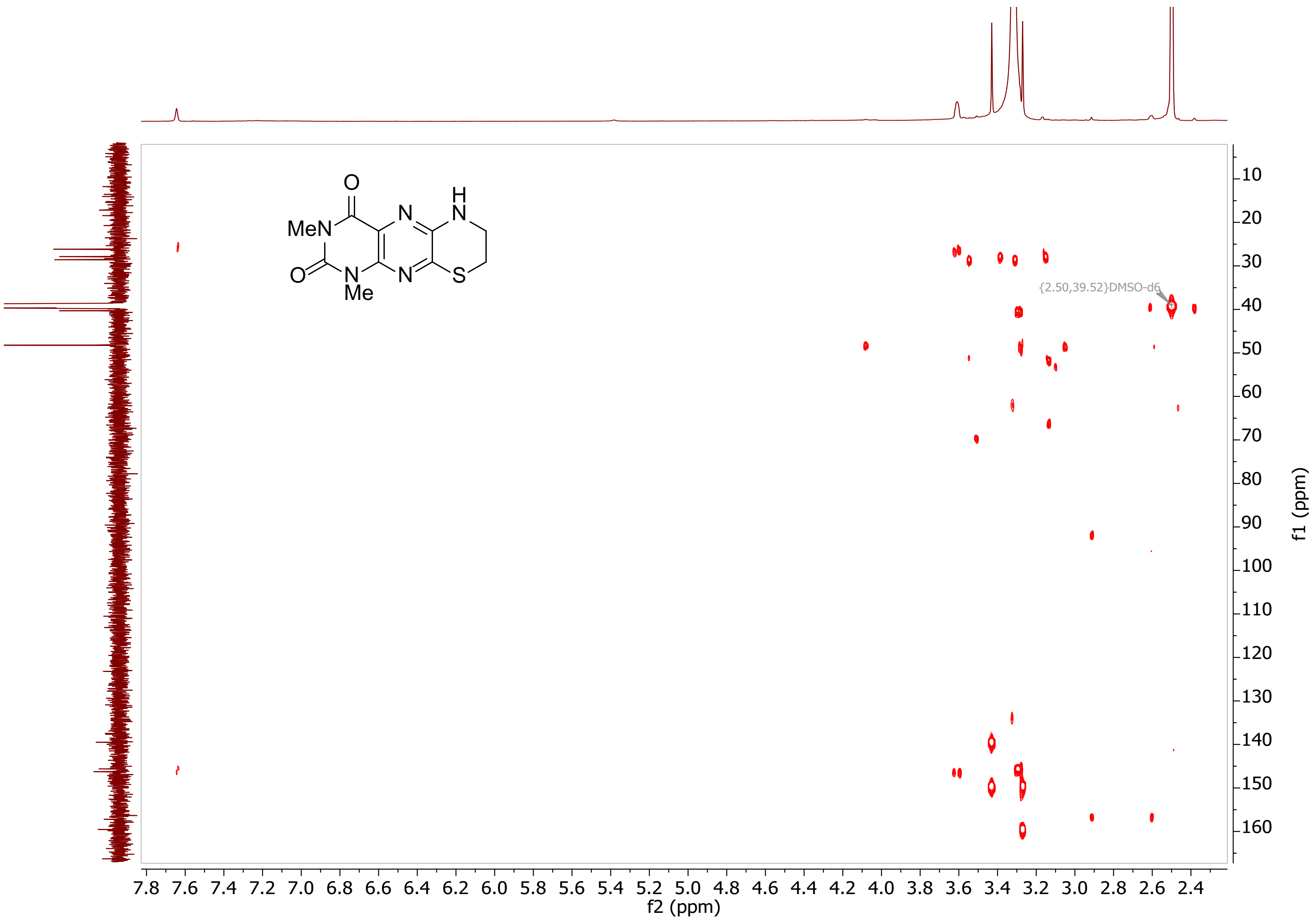

Figure S15: HMBC NMR spectrum (600 MHz) of tedaniophorbasin B (**2**) in DMSO-d_6.

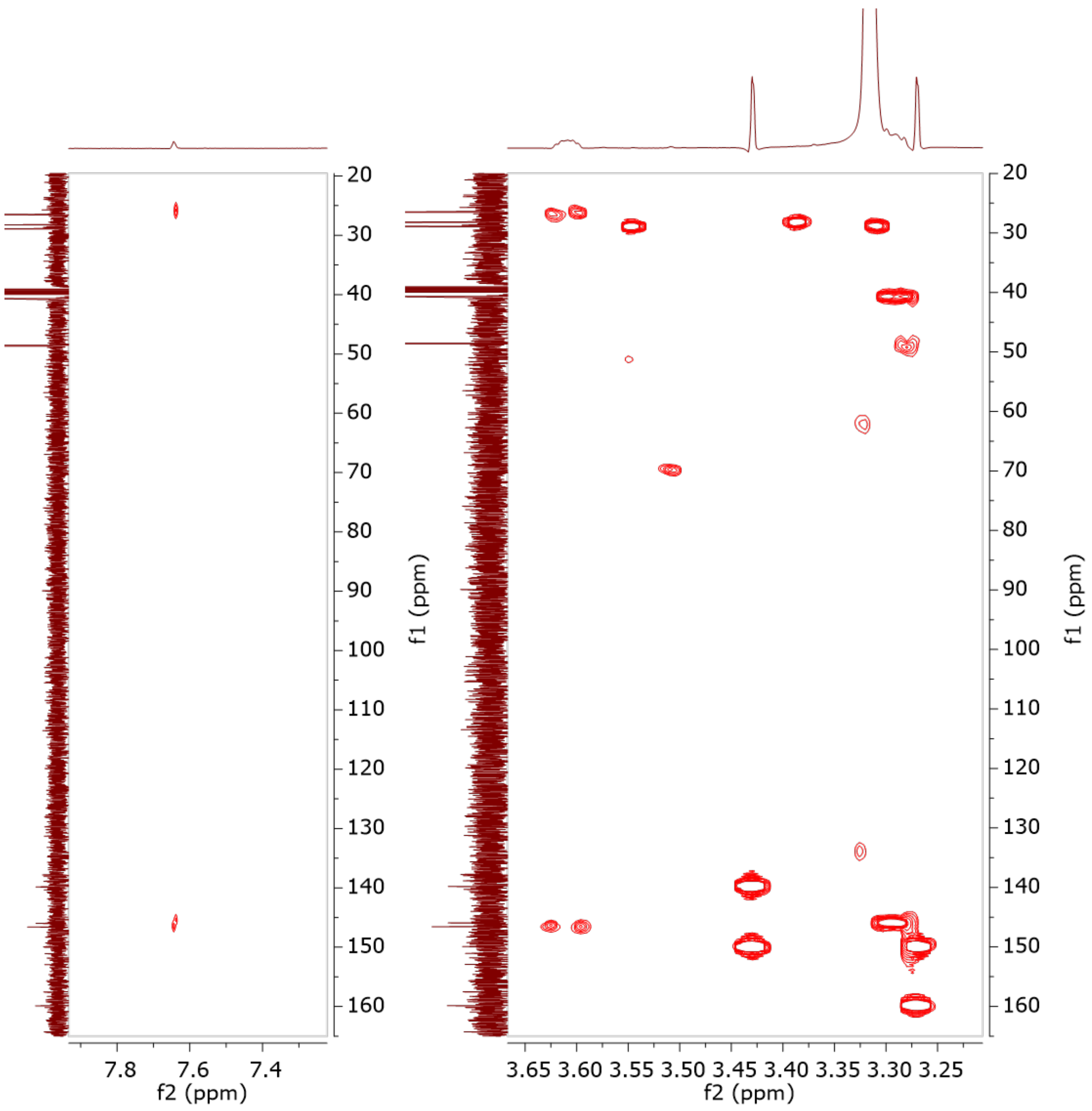

Figure S16: HMBC NMR spectrum (600 MHz) Expansions for tedaniophorbasin B (**2**) in DMSO-d_6.

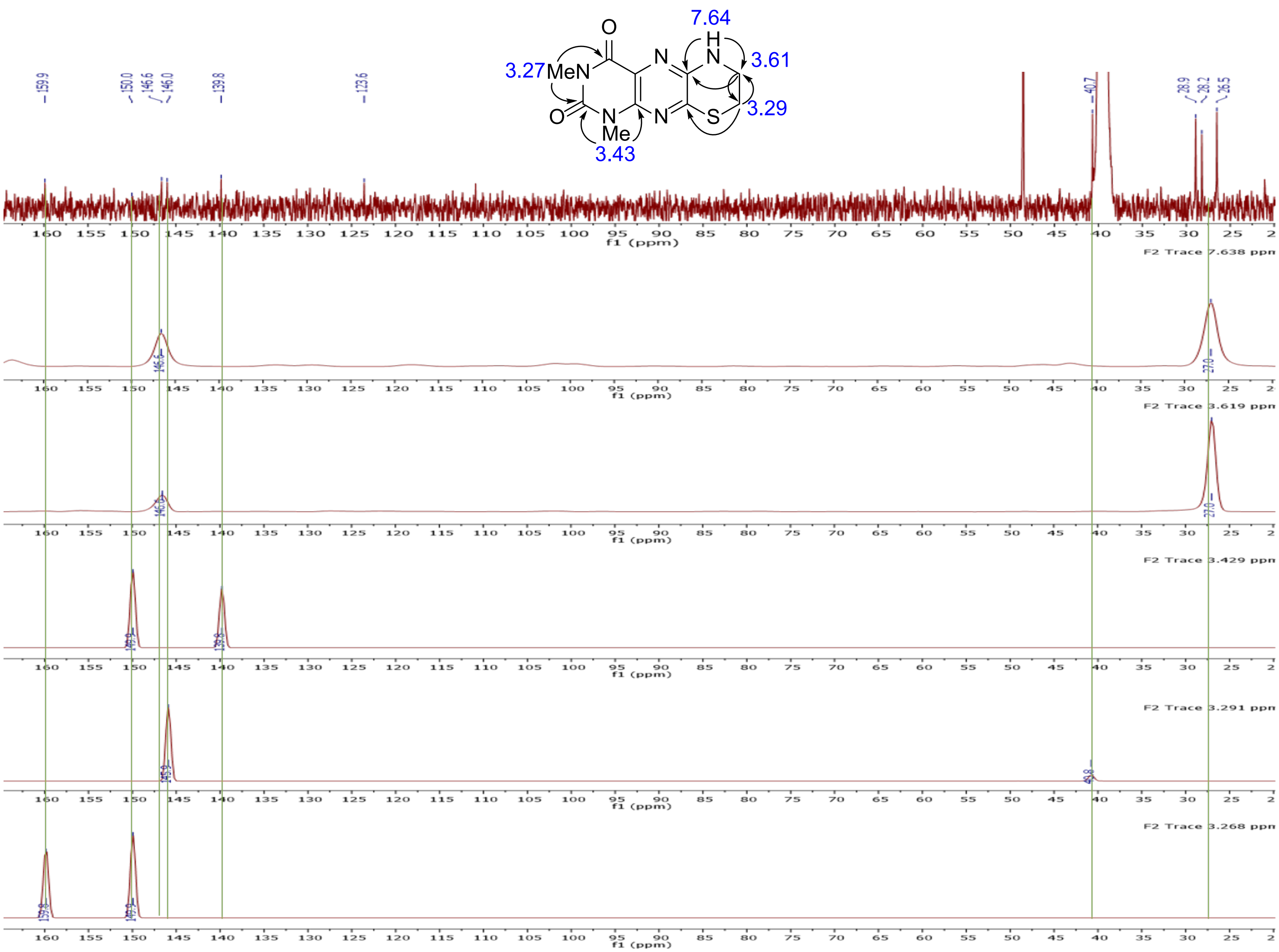

Figure S17: HMBC NMR Traces at each ^{1}H resonance chemical shift (600 MHz) of tedaniophorbasin B (**2**) in DMSO-d_6

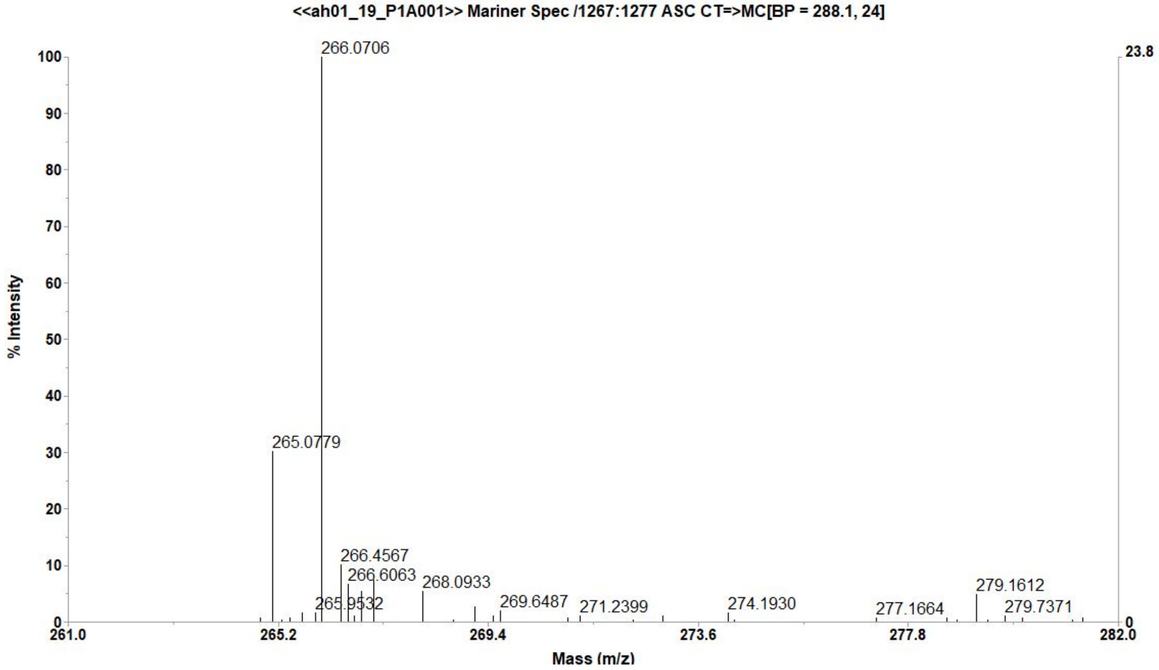

Figure S18: HRESIMS [M+H]$^+$ of tedaniophorbasin B (**2**)

#	MF	Monoisotopic mass	Ionization	*m/z*	PPM	mDa	unsaturation
1	$C_{10}H_{12}N_5O_2S$	266.0712	H^+	266.0706	-0.08	-0.02	7.5
2	$C_2H_8N_{11}O_5$	266.0710	H^+	266.0704	0.61	0.16	4.5
3	CH_2N_{18}	266.0710	H^+	266.0704	0.63	0.17	10
4	$C_{10}H_{20}NOS_3$	266.0707	H^+	266.0702	1.68	0.45	1.5
5	$C_{18}H_8N_3$	266.0718	H^+	266.0713	-2.53	-0.67	16.5

Calculated DFT (GIAO) ^{13}C NMR Chemical Shifts for 1a, 1a′, 2a and 2a′.

Table S1. Tedaniophorbasin A (1a) DFT vs Experimental ^{13}C NMR data for 1 (DMSO-d_6).

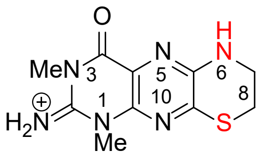

1a

Atom numbers	Experimental ^{13}C chemical shift	DFT Calcd. ^{13}C chemical shifts[3]	Absolute Error values
1 (N-CH₃)	31.3	28.1	3.2
2	151.0	150.0	1.0
3 (N-CH₃)	30.3	27.3	3.0
4	157.4	154.0	3.4
4a	120.6	124.2	3.6
5a	148.0*	150.0	2.0
7	40.6	40.3	0.3
8	26.3	25.1	1.2
9a	147.9*	148.5	0.6
10a	137.6	137.6	0.0

^{13}C MAE = 1.8

Table S2. Tedaniophorbasin A (1a′) DFT vs Experimental ^{13}C NMR data for 1 (DMSO-d_6).

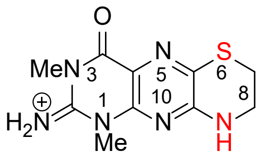

1a'

Atom numbers	Experimental ^{13}C chemical shift	DFT Calcd. ^{13}C chemical shifts[3]	Absolute Error values
1 (N-CH₃)	31.3	27.4	3.9
2	151.0	151.2	0.2
3 (N-CH₃)	30.3	27.0	3.3
4	157.4	151.7	5.7
4a	120.6	118.5	2.1
5a	147.9*	140.7	7.2
7	26.3	22.7	3.6
8	40.6	42.5	1.9
9a	148.0*	150.1	2.1
10a	137.6	144.2	6.6

^{13}C MAE = 3.7

***sDP4+ probability = tedaniophorbasin A (1a, proposed) 100% vs alternate (1a′) 0%[1]**

Table S3. Tedaniophorbasin B (2a) DFT vs Experimental ^{13}C NMR data for 2 (DMSO-d_6).

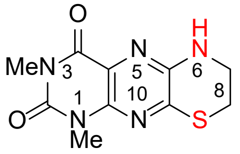

2a

Atom numbers	Experimental ^{13}C chemical shift	DFT Calcd. ^{13}C chemical shifts[3]	Absolute Error values
1 (N-CH₃)	28.9	29.4	0.5
2	150.0	149.4	0.6
3 (N-CH₃)	28.2	28.6	0.4
4	159.9	159.2	0.7
4a	123.6	124.6	1.0
5a	146.6	145.6	1.0
7	40.7	40.4	0.3
8	26.5	26.3	0.2
9a	146.0	141.8	4.2
10a	139.8	144.2	4.4

^{13}C MAE = 1.3

Table S4. Tedaniophorbasin B (2a') DFT vs Experimental ^{13}C NMR data for 2 (DMSO-d_6).

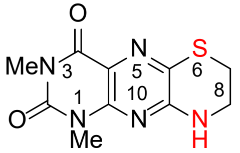

2a'

Atom numbers	Experimental ^{13}C chemical shift	DFT Calcd. ^{13}C chemical shifts[3]	Absolute Error values
1 (N-CH₃)	28.9	28.9	0.0
2	150.0	149.8	0.2
3 (N-CH₃)	28.2	28.4	0.2
4	159.9	157.8	2.1
4a	123.6	121.6	2.0
5a	146.0	134.3	11.7
7	26.5	23.5	3.0
8	40.7	42.1	1.4
9a	146.6	149.2	2.8
10a	139.8	148.8	9.0

^{13}C MAE = 3.2

***sDP4+ probability = tedaniophorbasin B (2a, proposed) 100.0% vs alternate (2a') 0.0%[1]**

1. Grimblat, N.; Zanardi, M. M.; Sarotti, A. M. Beyond DP4: An Improved Probability for the Stereochemical Assignment of Isomeric Compounds Using Quantum Chemical Calculations of NMR Shifts. *J. Org. Chem*. **2015**, *80*, 12526–125
